# Transplantation of retinal pigment epithelium and photoreceptors generated concomitantly via small molecule-mediated differentiation rescues visual function in rodent models of retinal degeneration

**DOI:** 10.1186/s13287-021-02134-x

**Published:** 2021-01-19

**Authors:** Harshini Surendran, Swapna Nandakumar, Vijay Bhaskar Reddy K, Jonathan Stoddard, Varsha Mohan K, Pramod K. Upadhyay, Trevor J. McGill, Rajarshi Pal

**Affiliations:** 1 Eyestem Research, Centre for Cellular and Molecular Platforms (C-CAMP), National Centre for Biological Sciences-Tata Institute of Fundamental Research (NCBS-TIFR) Campus, GKVK Post, Bellary Road, Bangalore, 560065 Karnataka India; 2 Casey Eye Institute, Oregon Health and Science University, Portland, OR USA; 3 National Institute of Immunology, New Delhi, India

**Keywords:** Induced pluripotent stem cells, Retinal pigment epithelium, Photoreceptor, Age-related macular degeneration, Retinitis pigmentosa

## Abstract

**Background:**

Age-related macular degeneration (AMD) is a result of degeneration/damage of the retinal pigment epithelium (RPE) while retinitis pigmentosa (RP), an inherited early-onset disease, results from premature loss of photoreceptors. A promising therapeutic approach for both is the replacement of lost/damaged cells with human induced pluripotent stem cell (hiPSC)-derived retinal cells.

**Methods:**

The aim of this study was to investigate the in vivo functionality of RPE and photoreceptor progenitor (PRP) cells derived from a clinical-grade hiPSC line through a unified protocol. De novo-generated RPE and PRP were characterized extensively to validate their identity, purity, and potency.

**Results:**

RPE expressed tight junction proteins, showed pigmentation and ciliation, and secreted polarization-related factors vascular endothelial growth factor (VEGF) and pigment epithelium-derived factor (PEDF). PRP expressed neural retina proteins and cone and rod markers, and responded to KCl-induced polarization. Transcriptomic analysis demonstrated an increase in the expression of mature retinal tissue-specific genes coupled with concomitant downregulation of genes from undesired lineages. RPE transplantation rescued visual function in RCS rats shown via optokinetic tracking and photoreceptor rescue. PRP transplantation improved light perception in NOD.SCID-rd1 mice, and positive electroretinography signals indicated functional photoreceptor activity in the host’s outer nuclear layer. Graft survival and integration were confirmed using immunohistochemistry, and no animals showed teratoma formation or any kind of ectopic growth in the eye.

**Conclusions:**

To our knowledge, this is the first demonstration of a unified, scalable, and GMP-adaptable protocol indicating strong animal efficacy and safety data with hiPSC-derived RPE and PRP cells. These findings provide robust proof-of-principle results for IND-enabling studies to test these potential regenerative cell therapies in patients.

**Supplementary Information:**

The online version contains supplementary material available at 10.1186/s13287-021-02134-x.

## Background

Age-related macular degeneration (AMD) is the primary cause of blindness in developed countries and the third leading cause of blindness globally [1]. Age poses a significant risk factor for AMD, and the prevalence of AMD is expected to rise given the increase in life expectancy. An estimated 288 million are likely to be afflicted with AMD by 2040 [2, 3].

AMD affects the macula and in advanced stages causes loss of central vision [4]. AMD can be classified into “dry or atrophic” and “wet or exudative” types. Dry AMD could be considered a precursor to wet AMD, and only 10–15% of the patients with AMD progress to wet AMD [1]. In the early stages, AMD is characterized by the loss of retinal pigment epithelium (RPE) and accumulation of extracellular aggregates, called drusen, that accumulate between Bruch’s membrane and the RPE layer [4]. The late stage is characterized by geographic atrophy, with degeneration of RPE and loss of photoreceptors that rely on RPE for trophic support. Wet AMD presents with choroidal neovascularization, where new blood vessels grow into the retina and leak blood and fluid leading to the destruction of the RPE and photoreceptors [1, 4]. While inhibiting vascular endothelial growth factor (VEGF) has shown promise in the treatment of wet AMD, there are currently no treatment modalities for atrophic AMD.

While AMD predominantly affects elderly people, retinitis pigmentosa (RP), a heterogeneous group of genetic disorders that affects 1 in 3000–7000 people, often appears in the early years. Patients with RP experience severe visual impairment by the age of 40–50 years [5]. Degeneration of rod photoreceptors with subsequent loss of cone photoreceptors is the hallmark of RP. Typical symptoms include night blindness and tunnel vision, eventually leading to complete blindness [5]. Phenotypic differences aside both eye disorders are progressive and result from degeneration of cells in the retina. Loss of vision lowers the quality of life and poses a significant financial and societal burden. Currently, there are no treatments available for degenerative eye disorders, and cell replacement strategies hold great potential. The benefits from autologous transplantation of RPE provide strong evidence in favor of cell replacement therapy [6, 7].

RPE plays a crucial role in the maintenance of photoreceptor function, and the death of RPE translates into the loss of photoreceptors and subsequent loss of vision [8]. Replacement of dead or dysfunctional RPE with that of stem cell-derived RPE is a promising alternative to treat AMD and some forms of RP. Preclinical and clinical trials are now underway to test the efficacy of pluripotent stem cell-derived RPE [9–12]. While treatment strategies focused on RPE replacement might prevent further deterioration, their role in vision restoration might be limited due to the lack of availability of off-the-shelf photoreceptors. Additionally, RPE replacement alone is not likely to provide preventative or restorative benefits in other eye disorders such as RP.

Here, we report a new unified method that allows the simultaneous generation of RPE and photoreceptor progenitors (PRP) from a single starting source of induced pluripotent stem cells (iPSCs). We exploit the capacity of bipotential retinal progenitors to form more than one cell type and provide a common platform that can be used to generate neurosensory retinal cells and the RPE, eliminating the need for devising separate methodologies. This approach is a step forward in enabling a more efficient and cost-effective route to cell replacement therapies. We provide extensive data describing the characteristics of both these iPSC-derived cell types, generated in unison, demonstrating pilot safety and efficacy in relevant diseased animal models.

## Materials and methods

### Reagents and assay kits

The basal media were DMEM/F12 (Thermo Fisher Scientific, Waltham, MA) and Minimum Essential Medium- alpha modified (MEMα) (Sigma-Aldrich, St. Louis, MO). The medium supplements were knock-out serum (KOSR), HEPES buffer, GlutaMAX, non-essential amino acids (NEAA), sodium bicarbonate, sodium pyruvate, and TrypLE select (Gibco, Thermo Fisher Scientific). The defined media were mTeSR™1, Accutase, and Neural Rosette Selection Reagent (Stem Cell Technologies, Vancouver, Canada). The growth factors and small molecules were IGF-1, IWR1, LDN193189 (Stem Cell Technologies), SB431542, and Y27632 (Tocris, Bristol, UK); hydrocortisone, taurine, tri-iodo-thyronine, and N1 supplement (Sigma-Aldrich). RNA Isolation kit (Qiagen, Hilden, Germany), Superscript™ II Reverse transcription kit and SYBR Green Master mix (Thermo Fisher Scientific).

### iPSC culturing, retinal induction, RPE, and PRP differentiation

All experiments using human iPSC (hiPSC) lines were conducted per the guidelines from the Indian Council of Medical Research (ICMR), Ministry of Health, Government of India, after obtaining approval from the Institutional Committee for Stem Cell Research (ICSCR), Eyestem Research Private Limited, CCAMP, NCBS-TIFR, Bangalore. The data shown here was generated using the TC-1133, the clinical-grade hiPSC line procured from Rutgers University Cell and DNA Repository (RUCDR) after signing the MTA with National Institute of Health (NIH), Bethesda, MD, USA, and maintained in feeder-free conditions [13]. Briefly, cells were cultured on human embryonic stem cell (hESC)-qualified Matrigel-coated (Corning, NY) plates in mTeSR medium (Stem Cell Technologies) with everyday media change. Cells were passaged at 1:4 to 1:6 ratio whenever they reached 80–90% confluency. A 1% Matrigel solution was used for all adherent cultures, and culture plates were coated with Matrigel solution overnight at 37 °C.

The approach used here to generate RPE and PRP was based on the previously reported RPE differentiation protocol [14]. iPSCs were plated in low attachment dishes to form aggregates called embryoid bodies (EBs). These EBs were maintained in mTeSR for 2 days and gradually exposed to differentiation induction media (DIM) and plated for propagation as adherent cultures in Matrigel-coated tissue culture plates. Post induction for 5 days, cultures were allowed to grow in a differentiation propagation medium (DPM). Between days 18 and 20, rosette populations were selected using Neural Rosette Selection Reagent (Stem Cell Technologies). The remaining non-rosette population was harvested using Accutase. Both cultures were separately plated onto Matrigel-coated plates in DPM. The rosette population was plated at a density of 1.5 × 10^6^ cells/10 cm^2^ to generate PRP cells. The non-rosette population was plated at 1.0 × 10^6^ cells/10 cm^2^ to generate RPE cells. After 2–3 days, the non-rosette population was gradually shifted to RPE maturation medium (RMM), whereas the rosette population was maintained in DPM.

### Transplantation of RPE cell suspension via trans-scleral subretinal injection in RCS rats

All animal procedures were approved and performed under the supervision of the Institutional Animal Care and Use Committee (IACUC) at Oregon Health and Science University, Portland. A total of 16 (8 male and 8 female) inbred pigmented Royal College of Surgeons (RCS-p+/Lav) rats with a minimum body weight of 25 g and age range of postnatal day 20–23 were used for the study. Animals were maintained on oral cyclosporine (210 mg/L) administered in the drinking water from 1 day prior to transplantation until the animals were sacrificed. RCS rats were weighed and sedated with a ketamine/xylazine (100/10 mg/kg) cocktail on post-natal day 21. Briefly, a small tunnel incision was made through the conjunctiva and sclera in the dorso-temporal region of the eye using a 30-gauge needle. A small glass pipette connected through microtubing to a 10-μL Hamilton syringe was then inserted into the subretinal space, and 2 μL of cell suspension (50,000 and 100,000 cells) was injected. The needle was held in place for a count of 10 before removal to limit efflux of cells. Successful injection was confirmed by manual visualization of the fundus and lack of abnormal or negative outcomes. One animal did not recover from anesthesia and died shortly after the injection of the cells. Half of the animals were sacrificed for histological study at P60 (*n* = 7) and the remaining animals at P90 (*n* = 8).

### Transplantation of PRP cell suspension via trans-corneal subretinal injection in NOD.SCID-rd1 mice

Approval for animal transplantation was obtained from the Institutional Animal Ethics Committee (IAEC) and Institutional Committee for Stem Cell Research (ICSCR) at the National Institute of Immunology, New Delhi. A total of 15 NOD.SCID-rd1 mice with a minimum body weight of 18 g and age range of postnatal days 28–42 were used for the study, and animals were kept at a 14-h light-10-h dark cycle [15]. Tropicamide ophthalmic solution (1%) was used to dilate the pupil and was dark-adapted for 30 min to attain maximum dilation, followed by anesthetization using ketamine (80–100 mg/kg) and xylazine (10 mg/kg) intraperitoneally. The mouse was placed in lateral recumbence, exposing the eye. The needle was inserted at 45° at the corneal-scleral intersection; 2 μL cell suspension (200,000 cells) of day 55 (*n* = 5) and day 75 (*n* = 5) PRP and vehicle (*n* = 5) was injected into the subretinal region. The animals were housed in individually ventilated cages and monitored for revival from anesthesia and ocular inflammation such as redness.

## Results

### EB treatment with small molecules promotes eye-field induction

**Fig. 1:**
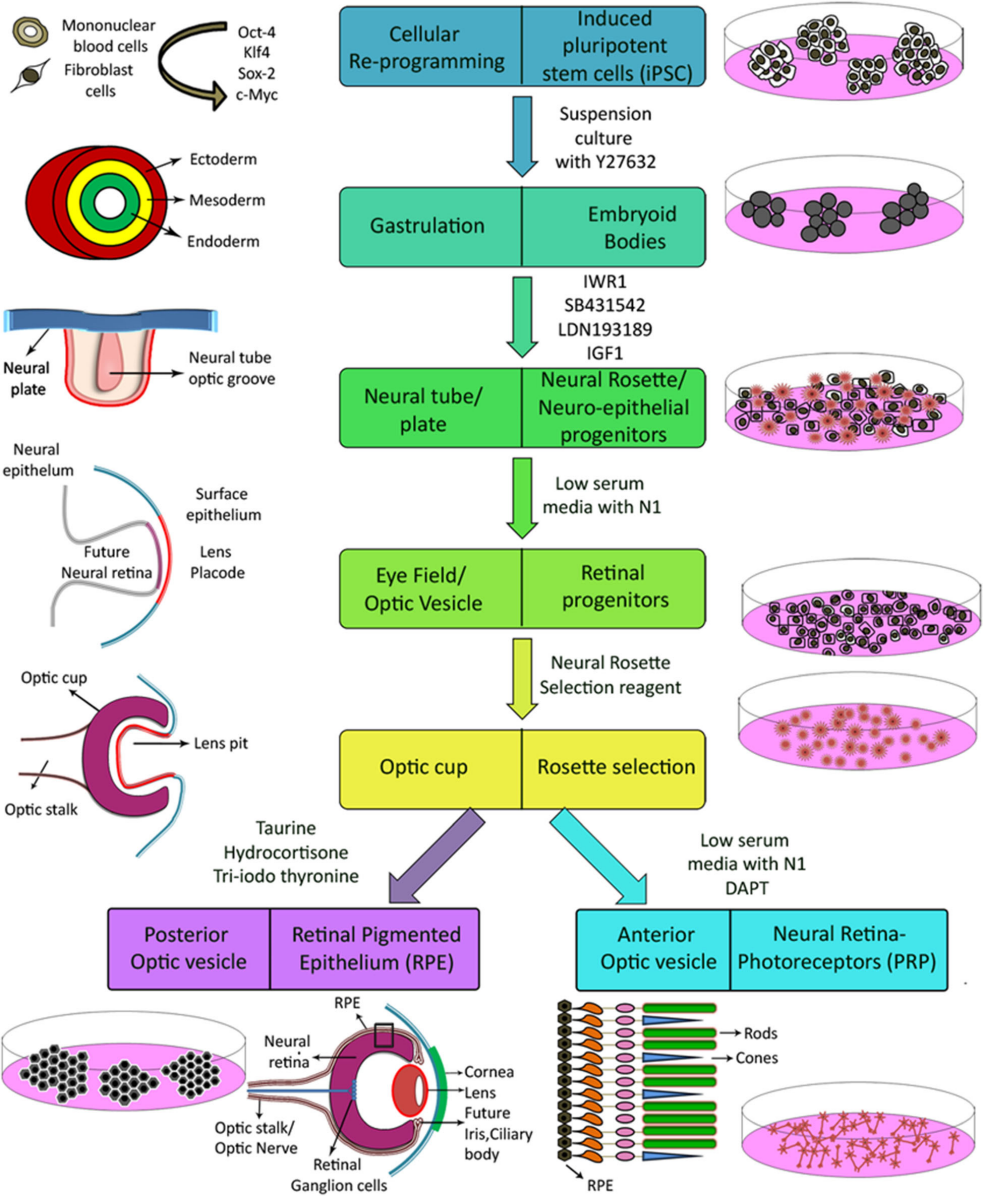
Schematic diagram depicts how our unified differentiation simulates the in vivo milestones of human retinal development—starting from the undifferentiated iPSC until the formation of mature RPE and PRP cells

The differentiation process used here requires the sequential addition of growth factors and small molecule cocktails, resulting in three major steps that recapitulate in vivo retinal development (Fig. 1) to obtain RPE and PRP.

**Fig. 2:**
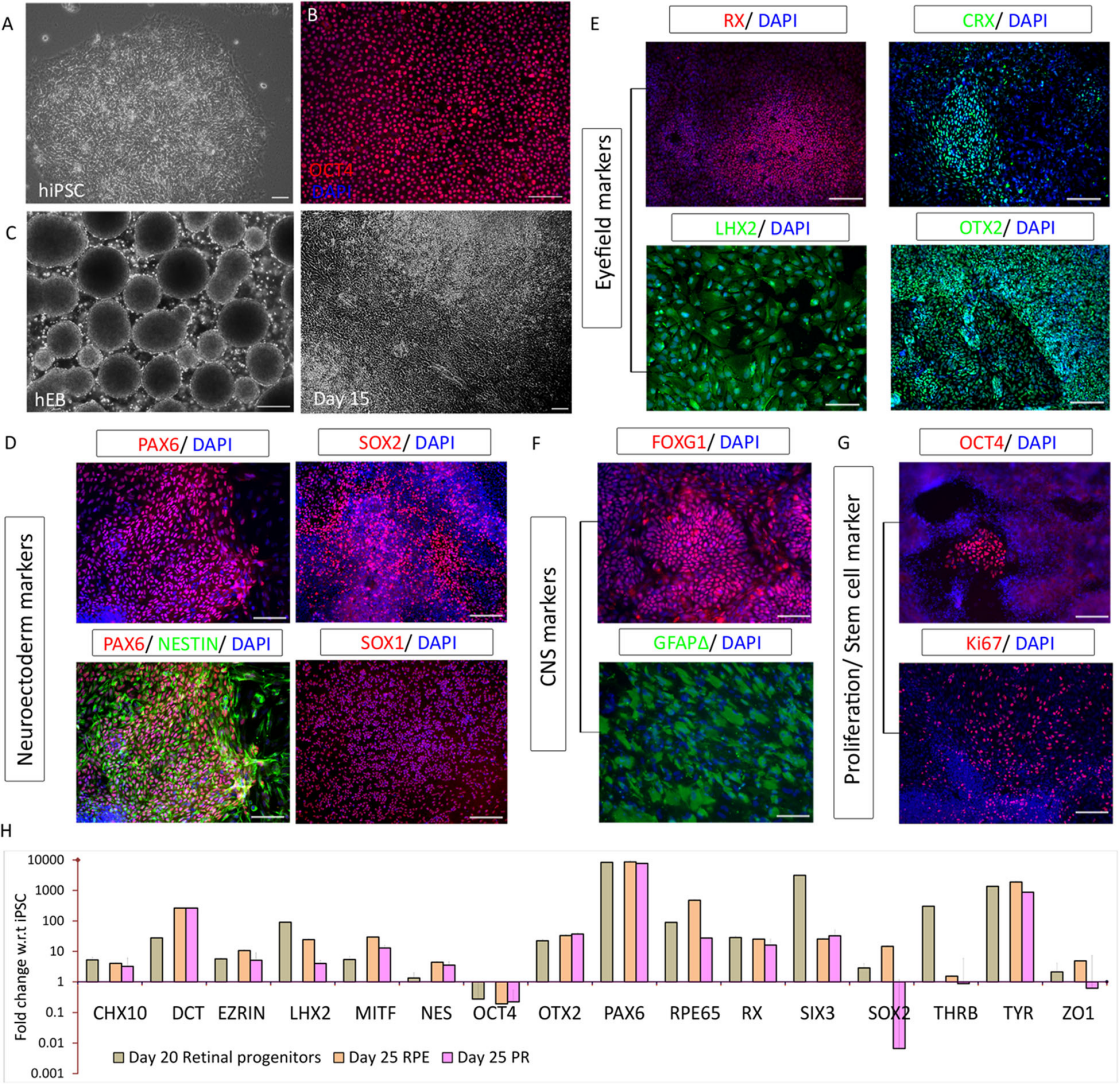
Induction of iPSC to retinal progenitors and their characterization. **a** TC-1133 hiPSC colony. **b** hiPSC expressing more than 95% OCT4. **c** Formation of embryoid bodies from iPSC and their induction to retinal progenitors. **d**–**g** Immunofluorescence images representing the protein expression at day 20 before selection. **d** Neuroectodermal markers PAX6, Nestin, SOX1, and SOX2. **e** Retinal commitment markers RX, LHX2, CRX, and OTX2. **f** Neural markers FOXG1 and GFAPΔ. **g** Expression of OCT4 and Ki67. **h** Gene quantification using real-time PCR of selected markers in day 20 retinal progenitors and day 25 RPE progenitors and photoreceptor progenitors after rosette selection represented as fold change with respect to the undifferentiated iPSC. Images and graphs are representative of a minimum of three independent experiments. Scale bars represent 100 μm

iPSC cultures were maintained as colonies (Fig. 2a). The iPSC cultures were > 95% positive for OCT4 (Fig. 2b) before the initiation of differentiation. During propagation, following exposure to DIM and plating of EBs, cells pattern themselves to form rosette-like structures surrounded by islands of epithelial population and some supporting cells (Fig. 2c).

Neuroectodermal lineage was induced using standard dual SMAD inhibition and addition of IGF1. Successful induction was confirmed based on the presence of neuroectodermal markers, PAX6, Nestin, SOX2, and SOX1 (Fig. 2d), and early retinal specification markers, RX, LHX2, CRX, and OTX2 (Fig. 2e). Proliferating neural precursor markers FOXG1 and GFAPΔ were also expressed at day 20 (Fig. 2f). Although OCT4 expression had dropped to < 15%, there was a significant population of cells positive for the proliferation marker Ki67 at the expansion stage (Fig. 2g) as expected.

At the optic vesicle stage, both distal and proximal domains are “bipotential” and capable of giving rise to either neuroretina or RPE [16]. Under the influence of the overlying surface ectoderm, the proximal domain acquires an RPE identity, and the distal domain develops into neuroretina. Quantitative gene profiling of the bipotential retinal progenitors and the separated RPE and PRP progenitors revealed differences in the gene expression patterns. The array of retinal progenitor genes was composed of early neuroectoderm and neuroretina genes, with detectable expression from day 20 progenitor stage (Fig. 2h). There was a significant upregulation of genes required for RPE and PRP fate commitment that was exclusive to non-rosette and rosette fractions, respectively (Fig. 2h). This gene profiling data confirms both successful eye-field induction and the efficiency of our rosette pickup process in yielding desired retinal cell types.

### Commitment of optic vesicle cells into RPE

**Fig. 3:**
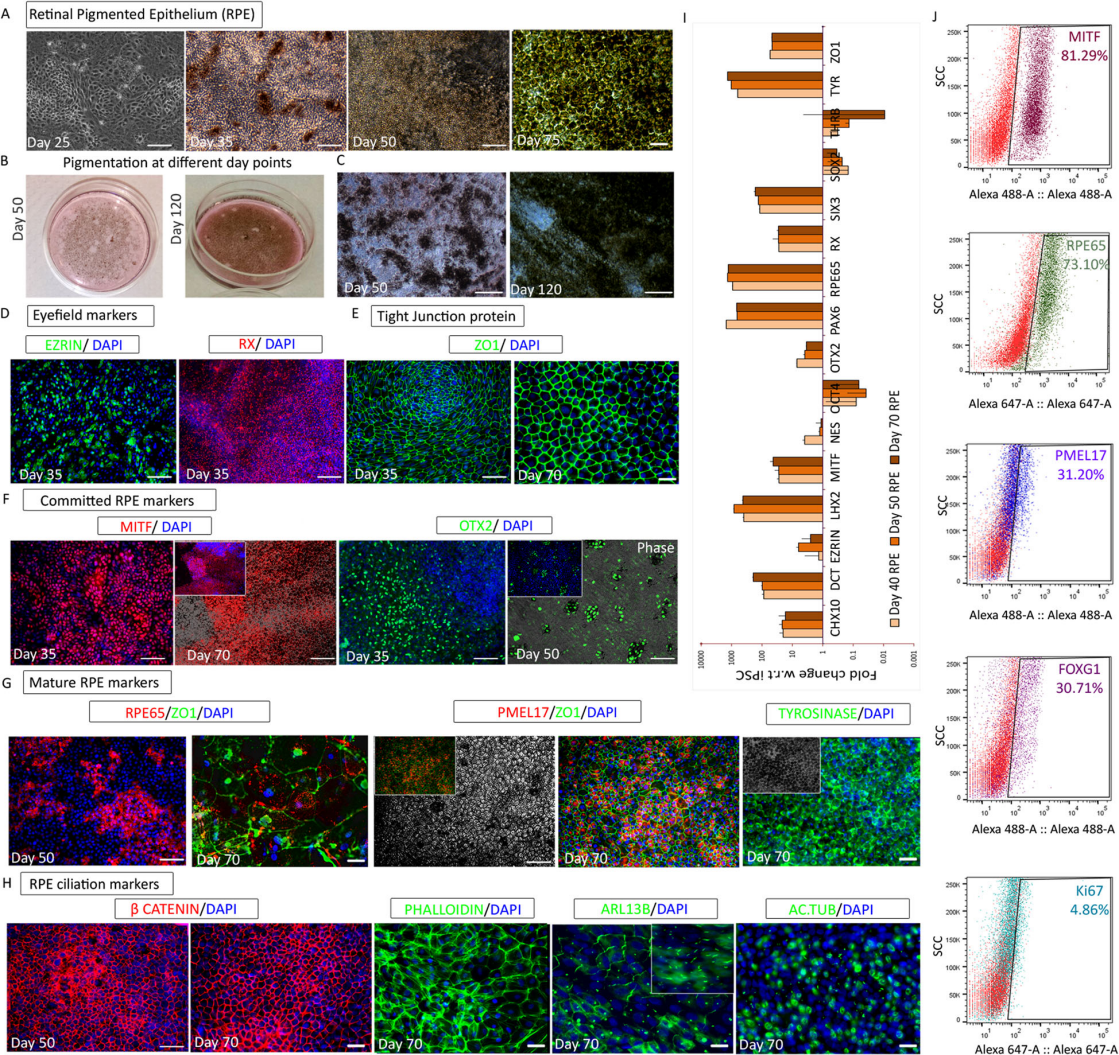
Differentiation and characterization of the retinal pigment epithelium (RPE). **a**–**c** Bright-field images during the course of differentiation. **a** Epithelial population starting to show pigmentation and typical hexagonal morphology. **b** RPE culture plates showing the incremental levels of visible pigmentation at progressive day points. **c** Morphology of pigmenting RPE cultures at different day points. **d**–**h** Immunofluorescence images representing the protein expression during different stages of RPE differentiation. **d** RPE progenitor markers Ezrin and RX at day 35. **e** Tight junction protein ZO1 on day 35 and day 70. **f** RPE commitment factor MITF at day 35 and day 70; RPE maintenance factor OTX2 at day 35 and day 50. **g** Mature RPE markers RPE65 at day 50 and day 70 and PMEL17 and Tyrosinase at day 70. **h** RPE ciliation markers β-catenin at day 50 and day 70 and Phalloidin, ARL13B, and acetylated tubulin at day 70. **i** Gene quantification profile using real-time PCR of selected markers represented as fold change in 40-, 50-, and 70-day RPE with respect to undifferentiated iPSC. **j** Flow cytometry analysis of MITF, RPE65, PMEL17, FOXG1, and Ki67 in day 70 RPE. Images and graphs are representative of a minimum of three independent experiments. Scale bars represent 100 μm

The selected non-rosette fraction was gradually switched to RMM. Cells were grown in 5% serum until they started pigmenting and maintained thereafter in 2% serum media (Fig. 3a). Pigmentation began at days 35–40 and continued to expand both in terms of intensity and the number of pigmenting colonies, with brown/black pigmentation eventually visible to the naked eye (Fig. 3b, c). RPE precursors expressed the cytoplasmic marker Ezrin and retinal homeobox transcription factor RX at day 35 (Fig. 3d). As expected, neuroretina markers PAX6, Nestin, and CRX (Figure S1A) and neural stem cell markers SOX2 and GFAPΔ had reduced at day 35, compared to day 20 retinal progenitors (Figure S1B). The strong expression of tight junction protein ZO1 (Fig. 3e) and the staining pattern confirmed the typical hexagonal morphology of the RPE cells as they reached day 70. The microphthalmia-associated transcription factor MITF (Fig. 3f) was detected as early as day 25 and continued to be expressed throughout the RPE differentiation. OTX2 expression which was detected at day 35, was restricted only to pigmenting colonies by day 50 (Fig. 3f). Visual cycle protein RPE65 appeared at day 50 and changed to distinct granular deposits by day 70 (Fig. 3g). Premelanosome protein PMEL17 and rate-limiting melanin biosynthesis enzyme, Tyrosinase, were both detected in fully pigmented regions of RPE cultures (Fig. 3g). The β-catenin localization varied between day 50 and day 70, indicating a change in the Wnt pathway status (Fig. 3h). The onset of ciliation, reported to be concomitant with Wnt pathway inactivation [17], was confirmed by the presence of cilia markers ARL13B and acetylated tubulin in the RPE cells (Fig. 3h). These cells also expressed high levels of filamentous actin as evidenced by phalloidin staining (Fig. 3h).

RPE-specific gene expression was ascertained at different stages (days 40, 50, and 70) using quantitative PCR (qPCR) (Fig. 3i). While *OCT4*, *SOX2*, and *THRβ* expression was found to be completely downregulated; the expression of pigmentation-related genes like *DCT* and *SIX3* gradually increased over time. RPE markers *MITF*, *ZO1*, *RPE65*, and *Tyrosinase* showed consistent expression, whereas the expression of progenitor and PRP markers decreased. mRNA sequencing data for the same set of genes, represented as heat maps, confirmed the qPCR results (Figure S1E). The percentage of cells expressing RPE-specific markers was further assayed using flow cytometry. More than 70% of the cells expressed MITF and RPE65 and about 30% of the cells expressed PMEL17 (Fig. 3j). Given that retinal cells originate from the neuroectoderm, we anticipated the presence of some neural cells in our early-stage RPE cultures, and about 30% of the cells were positive for FOXG1 (Fig. 3j). These day 70 FOXG1 cells were not proliferative as they were not positive for Ki67. However, a subset of MITF-positive cells was positive for Ki67 (Figure S1D), indicating proliferating RPE cells. There were no contaminating pluripotent cells in the RPE population demonstrated by the complete downregulation of OCT4 through qPCR (Fig. 3i) and immunostaining (Figure S1C). Enrichment of a cluster of genes associated with the visual cycle, pigmentation, and tight junction cell adhesion factors in RPE was evident from qPCR and sequencing data (Figs. 3i and 5i). The significant upregulation of adult and fetal RPE genes in mature RPE and RPE progenitors, respectively (Figure S5D, E), definitively confirms the RPE-specific signature of these cells. Additionally, neuroectoderm genes were silenced over time along with the complete downregulation of retinal ganglion genes in RPE (Fig. 5h; Figure S1A,B).

RPE closely interacts with immune cells through an array of secretory signaling molecules with distinct physiological roles. Vascular endothelial growth factor (VEGF) is secreted in response to oxidative stress due to the accumulation of glycation end products in Bruch’s membrane and plays a role in angiogenesis [18]. Pigment epithelium-derived growth factor (PEDF) protects the neurons against apoptosis and stabilizes the choriocapillaries, acting as an anti-angiogenic factor in proliferating endothelial cells [18]. Levels of VEGF and PEDF secreted from our RPE cultures were assayed, with iPSC and ARPE-19 as negative and positive controls, respectively. Both live and cryopreserved RPE cells at terminal day point secreted significantly higher amounts of VEGF and PEDF than ARPE-19 (Fig. 6a). Although no further enrichment steps were employed, the unambiguous presence of key markers and VEGF and PEDF secretion confirms the robust de novo generation of RPE. Therefore, day 70 RPE cultures that were used for in vivo studies largely comprised cells expressing RPE-specific signatures with some supporting cells of the retinal lineage.

### Derivation of PRPs through specification of the neuroretina

**Fig. 4:**
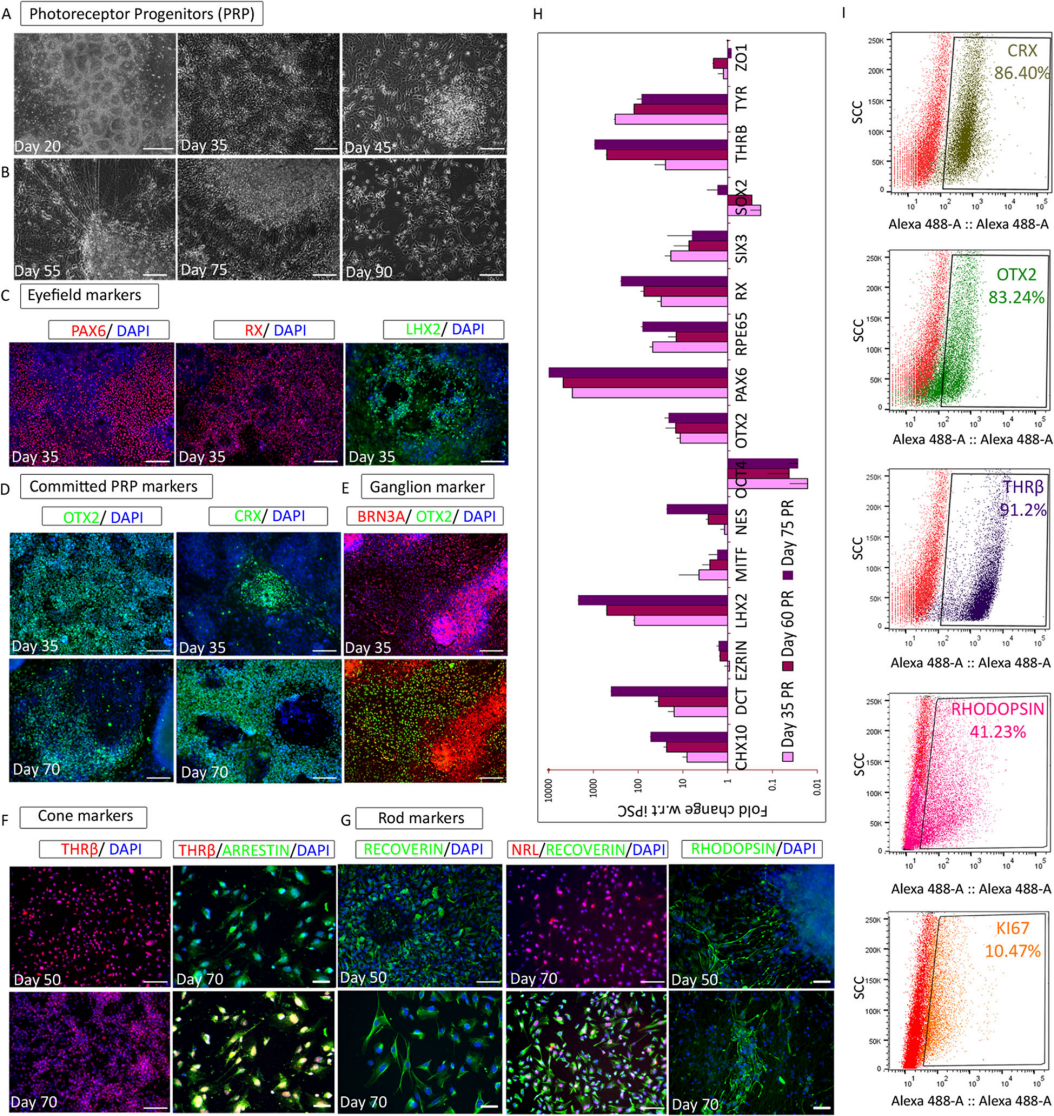
Propagation and characterization of photoreceptor progenitors (PRP). **a**, **b** Bright-field images during the course of differentiation. **a** Rosette population selected for expanding neuroretinal cells. **b** Formation of contact synapses in photoreceptors. **c**–**g** Immunofluorescence images representing the protein expression during stages of PRP differentiation. **c** Photoreceptor progenitor markers PAX6, RX, and LHX2 at day 35. **d** Photoreceptor commitment factors OTX2 and CRX at days 35 and 70. **e** Retinal ganglion marker BRN3A at day 35. **f** Cone cell markers THRβ at days 50 and 70 and visual arrestin at day 70. **g** Rod cell markers Recoverin and Rhodopsin at days 50 and 70 and NRL2 at day 70. **h** Gene quantification profile using real-time PCR of selected markers represented as fold change in 35-, 60-, 75-day RPE with respect to undifferentiated iPSC. **i** Flow cytometry analysis for CRX, OTX2, THRβ, Rhodopsin, and Ki67 in day 70 PRP. Images and graphs are representative of a minimum of three independent experiments. Scale bars represent 100 μm

Tightly packed rosettes that appeared after exposure to DPM were selected during rosette pick up and replated. The rosette-rich fraction was maintained in DPM to induce neuroretina formation that eventually gives rise to PRPs (Fig. 4a). Around day 50, cells with dendritic projections considered to be PRPs that connected with each other were observed. These PRPs on further maturation convert to photoreceptors, including rods and cones. During differentiation, a dense and complex network between the cells was noted (Fig. 4b). The expression of PAX6, RX, and LHX2 observed at day 20 remained consistent throughout the differentiation (Fig. 4c). OTX2 expression was observed at day 30 and showed uniform expression until the final stage, unlike in RPE (Fig. 4d). The expression of the cone-rod homeobox transcription factor, CRX, which is important for photoreceptor differentiation [19], was observed from day 35 to day 70 (Fig. 4d). Both MITF and ZO1 showed weak expression at day 35, while ZO1 started to disappear after day 50 (Figure S2A). At day 35, a subset of cells was positive for SOX1, Nestin, and GFAPΔ; FOXG1 was detected in most of the cells (Figure S2B). By day 70, FOXG1 expression was only detected in 30% of the cells, but there was no GFAPΔ expression (data not shown).

Typically, neuroretina cultures also consist of ganglion cells, along with cone and rod cells. As expected, the retinal ganglion marker BRN3A started to co-express with OTX2 at day 35 (Fig. 4e). The expression of cone cell marker THRβ was detected from day 50, and at day 70, it co-expressed with visual arrestin, which plays a critical role as a molecular switch in rod and cone cells during phototransduction (Fig. 4f). Recoverin is a retinal calcium-binding protein, which acts upstream of rhodopsin regulating its phosphorylation. Both proteins expressed comparably with Recoverin showing a more intense staining pattern (Fig. 4g). Additionally, the expression of both proteins was restricted to distinct axon-like projections at day 70 (Fig. 4g). The transcription factor NRL, which is important for rod cell specification, communicates with CRX to regulate rhodopsin transcription and controls photoreceptor survival [20]. As anticipated, NRL appeared along with Recoverin in our day 70 cultures (Fig. 4g). There was minimal to no OCT4 expression, while a low level of Ki67 was seen in our PRP cells (Fig. 3i, Figure S2C).

**Fig. 5:**
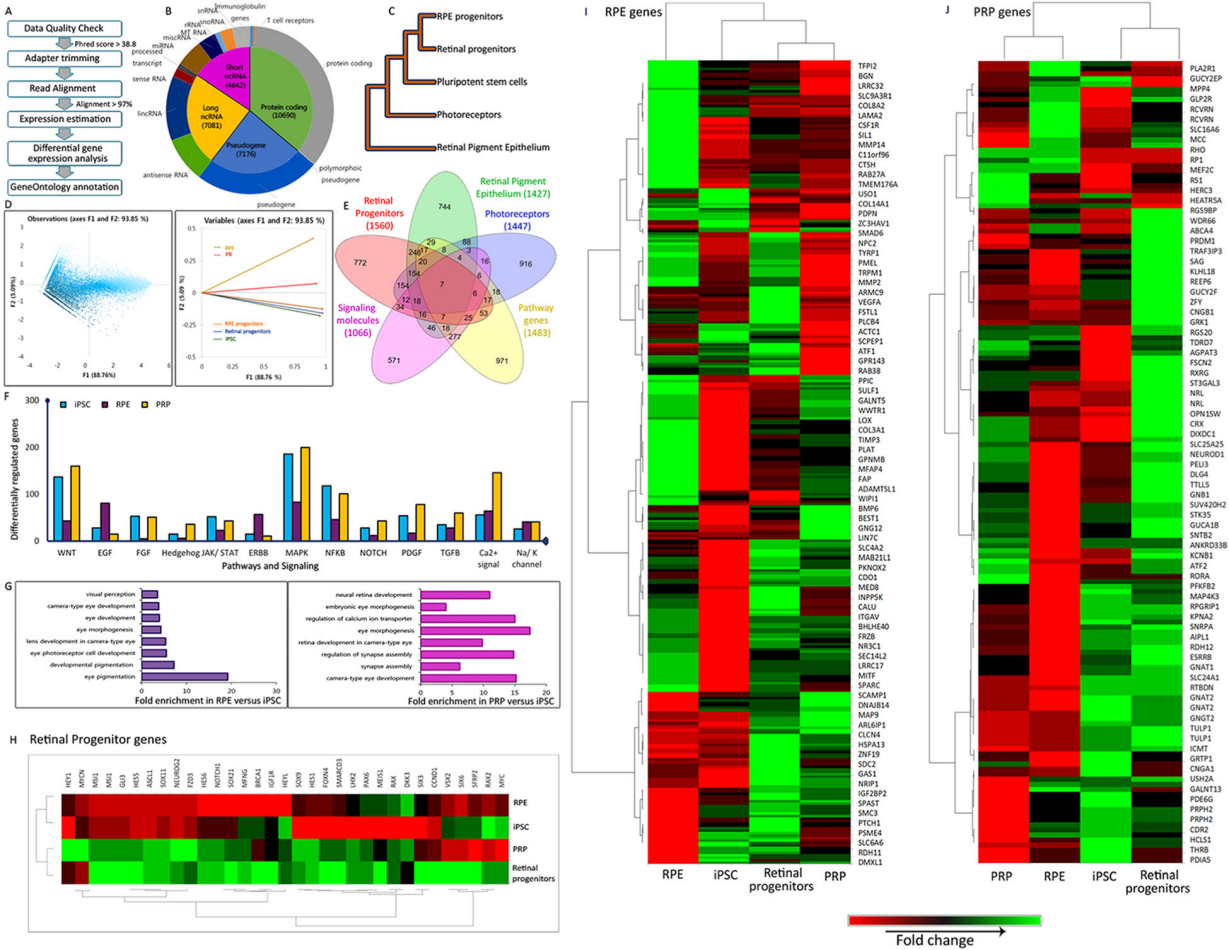
RNAseq data analysis and interpretation. **a** Flow chart of RNAseq workflow and bioinformatic analysis. **b** Pie chart showing the distribution of genes picked up by samples, categories further explained by doughnut chart. **c** Hierarchical clustering of samples using the UPGMA method. **d** Principal component analysis showing the relation between active variables. **e** Five sample Venn diagrams of differentially expressed genes from retinal progenitors, RPE, and PRP; significantly expressed pathway genes and signaling molecules. **f** Column graph showing the regulation of various pathways in iPSC, RPE, and PRP samples. **g** Gene Ontology and fold enrichment analysis for RPE and PRP. **h**–**j** Heat map shows clustering and differential expression of key genes from RNAseq data across iPSC, day 20 retinal progenitors, and day 70 RPE and PRP. **h** Retinal Progenitors. **i** RPE signature genes. **j** Rod and cone-specific PRP genes. Heat map colors: red to green through black

PRP-specific gene expression was checked at various stages (days 35, 60, and 75) using qPCR (Fig. 4h). *CHX10*, *PAX6*, *LHX2*, and *RX* were significantly upregulated along with *BRN3A*, *OTX2*, *Recoverin*, and *THRβ*. As predicted, *MITF* and *Tyrosinase* were significantly downregulated. We also noticed the complete downregulation of *OCT4* and *ZO1* with minimal expression of *SOX2* at day 75 (Fig. 3h). Further, flow cytometry analysis confirmed that > 80% of the cells were positive for key PRP proteins including CRX, OTX2, and THRβ (Fig. 4i). Marker profiles were confirmed using mRNA sequencing data, which also showed upregulation of neuroretina genes (Fig. 5j; Figures S1E, S2D). By day 50, our PRP cultures almost exclusively comprised cells of the neuroretina, which was confirmed based on the expression profiling of key PRP markers. Further, KCl-induced depolarization was observed with day 55 PRPs (Figure S2E), and cells at day 55 and at day 75 were used for transplantation studies.

### Molecular signature of iPSC-derived RPE and PRP

We generated global RNA expression profiles of cells at different stages of retinal differentiation and compared them with those of iPSCs. The data was quality checked, and the average base quality Phred score was more than 38.8 with > 97% read alignment (Figure S4A). Unwanted sequences were removed, and the paired-end reads were aligned to the reference human genome GRCh37/hg19 from the UCSC database (Fig. 5a). We distinguished the gene types into coding/non-coding genes and then found 37% of the population to be protein-coding genes (Fig. 5b). Hierarchical cluster analysis and principal component analysis (PCA) were performed to evaluate the similarities or differences in the gene expression patterns among samples at various stages of differentiation. The RPE and retinal progenitors were clustered together, and all other samples were clustered separately (Fig. 5c). As expected, a combined variance of 93% (F1 vs. F2) indicated that the gene expression profiles of RPE and PRPs were spaced reasonably away from the starting iPSC population. The variance in samples increased with the progress in differentiation (Fig. 5d).

Normalized read count was generated via log transformation and represented as fold change in comparison with iPSC. These logarithmic (fold change) values were found to be normally distributed (data not shown). Those genes that were found to be 2 standard deviations away from the mean were considered statistically significant. We then analyzed the overlap in genes across retinal progenitors, RPE, and PRPs and found that a majority of the genes had no overlap indicating cell-specific expression profiles (Fig. 5e, Figure S4E). The grouping of neuroretina genes showed strong expression in retinal progenitors and PRP while only very low expression in RPE cells (Fig. 5h–j). Likewise, the overlap in signaling molecules and pathways across the three populations was minimal (Fig. 5f, Figure S4C, D, S5F). Comparisons of pathways involved in iPSCs, RPE, and PRPs confirmed differential regulation. Notably, the calcium signaling pathway was significantly upregulated in PRPs (Fig. 5f). Gene Ontology (GO) enrichment analysis was performed using the GO Consortium. The upregulated genes for retinal progenitors, RPE, and PRPs belong to the pathways related to eye development and function, identified as fold enrichment (Fig. 5g, S4B).

Genes associated with the development of the central nervous system were detected in retinal progenitors but were largely switched off at later stages of differentiation. A similar trend was observed for markers of other germ layers (Figure S5B, C). This in-depth expression profiling of RPE and PRPs validated the differentiation strategy used here, and the reproducibility of this method in other PSC lines including one of our in-house-generated iPSC line was also confirmed (Figure S3).

### RPE transplantation delayed the loss of visual acuity in the RCS rat

**Fig. 6:**
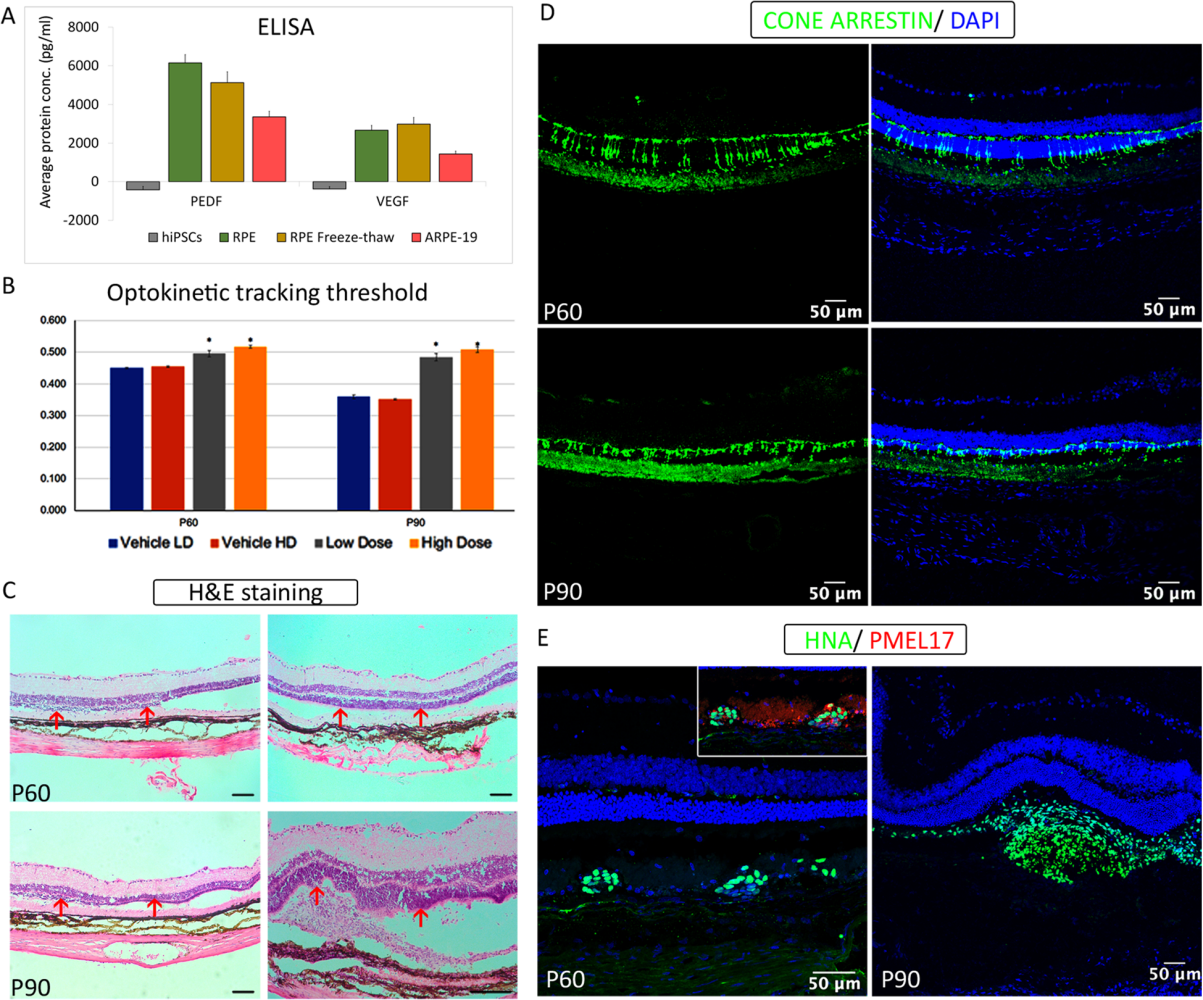
In vivo efficacy studies of RPE cells after transplantation into animal models. **a** ELISA-based quantification of polarization proteins secreted in in vitro culture supernatants—PEDF and VEGF in day 70 RPE running cells and freeze-thawed cells; hiPSCs and ARPE-19 cell line was used as negative and positive controls, respectively. **b**–**e** Functional and molecular outcome of RPE transplantation in RCS rats. **b** Optokinetic tracking thresholds measured from all animals at P60 and P90. Asterisks indicate statistical significance between cell-treated and BSS+-injected controls. **c** Histological evaluation using H&E staining data at P60 and P90. **d** Cone arrestin staining in the retina sections at P60 and P90. **e** Immunofluorescence images show the presence of transplanted cells detected using a human nuclear marker (HNA) co-stained with PMEL17 indicating graft survival and engraftment. Scale bars represent 50 μm

On confirming the molecular signatures of the de novo-generated RPE and PRPs, we proceeded to test their efficacy in rodent models. For RPE transplantation, we used the RCS rats with a recessively inherited mutation in the *MERTK* gene, which codes for receptor tyrosine kinase. This mutation prevents phagocytosis of photoreceptor outer segments by RPE, resulting in retinal degeneration. RCS rats are a commonly used model for RPE transplantation studies. RPE cells [50,000 (low dose) and 100,000 (high dose)] were successfully delivered into the subretinal space of all RCS rats on P21 (Figure S6A), and effective bleb formation was seen. The novel surgical approach (trans-scleral subretinal injection) employed here was developed to provide consistent delivery of cell suspension into the subretinal space without cell leakage into the vitreous [21]. One animal did not recover from anesthesia at the time of injection and was removed from the study. Optokinetic tracking (OKT) thresholds were measured through both eyes of each animal on post-natal days 60 and 90. There was no significant difference between the high- and low-dose cell-treated eyes at either age. However, there was a significant rescue of OKT thresholds in both high- and low-dose cell-treated eyes, compared with vehicle-injected controls (Fig. 6b). OKT values declined in vehicle-injected control eyes from P60 to P90, as anticipated. However, no such decline in the OKT values was observed in both the high- and low-dose cell-treated eyes. This rescue, despite degenerating controls, emphasizes the strength of the rescue effect. It should be noted that the average values of the OKT thresholds in cell-treated eyes include those animals that were observed later as having no remaining engrafted cells as assessed using immunohistochemistry. This likely led to less than optimal photoreceptor rescue in those animals, potentially reducing the maximum benefit of the transplanted iPSC-derived RPE cells.

### RPE maintain the functional and structural integrity of the host retina for extended periods

All eyes from surviving animals in the study (*n* = 15) were processed successfully for histological study. After cryosectioning and staining, the outer nuclear layer thickness (ONL) was measured (Figure S6B) as the primary indicator of photoreceptor rescue, and representative images of vehicle- and cell-injected tissue sections at P60 and P90 are shown (Fig. 6c). At P60, cell-treated eyes (temporal region) had significantly thicker ONL measurements than untreated areas of the same eye. Interestingly, although injection of BSS+ resulted in a modest level of photoreceptor protection in the retina, this was not comparable to the rescue achieved after RPE transplantation. However, by P90, the rescue effect of the BSS+ had waned, and rescue was only seen in the eyes that received RPE transplants, suggesting that BSS+ had a transient effect and that prolonged rescue could only be achieved with RPE cells. At P90, cell-treated retinas had significantly thicker ONL than untreated regions of the same eye and BSS+-treated eyes. Low- and high-dose cell-treated groups were combined for quantification of retinal thickness and cone counts (Figure S6B), eliminating the high variability and low number, to increase statistical power so these results could be a closer representation of the clear behavioral and histological preservation. These results indicated that the cell-treated regions of the retina provided significant preservation of photoreceptor nuclei, consistent with the rescued OKT thresholds.

Immunohistochemical analysis based on HNA and PMEL17 co-staining revealed the presence/survival of transplanted RPE cells at both the sacrificial ages (Fig. 6e). However, the number of surviving cells decreased with increasing age, and transplanted cells were not detected in all animals. In some of the animals in which no human cells could be identified, there was an influx of macrophages at areas of photoreceptor rescue that lacked a debris zone—indicative of where the transplanted cells resided before the atrophy of the graft. Evidence of graft rejection was seen in 2 out of 16 animals. In a few, but not all cases, we observed a small proportion of the transplanted human cells to be positive for both the human nuclear marker antigen and Ki67 (Figure S6C). It should be noted that the level of proliferation was low, and no potential tumorigenic outgrowths were observed. Cone arrestin was stained, and cone photoreceptors were significantly preserved with better morphology in the cell-treated (temporal) region of the eye, compared with the untreated (nasal) region of the same eye (Fig. 6d, S6B).

### PRP transplantation resulted in detectable electrical responses in NOD.SCID-rd1 mice

NOD.SCID-rd1 mouse, developed in NII, New Delhi [15, 22], is a double knockout model (*PDE6B−/− PRKDC−/−*) that mimics RP with slow-progressing photoreceptor degeneration. The model is bereft of photoreceptors by P28 and does not have functional adaptive immunity. Four-week-old mice (*n* = 5 per group) and of any gender (randomly selected) received either day 55 PRP (PRP55) or day 75 PRP (PRP75), while sham animals received only the vehicle (Figure S6D). Both eyes received 200,000 cells in the subretinal region, and no signs of ocular trauma were observed. Following transplantation, visual acuity and electro-physiological functions were assayed using behavioral apparatus and electroretinography (ERG), respectively. ERG stimulus and behavioral indicators were measured at an interval of 15 days post-first recording, wherein the first measurement was taken at day 2 post-transplantation.

**Fig. 7:**
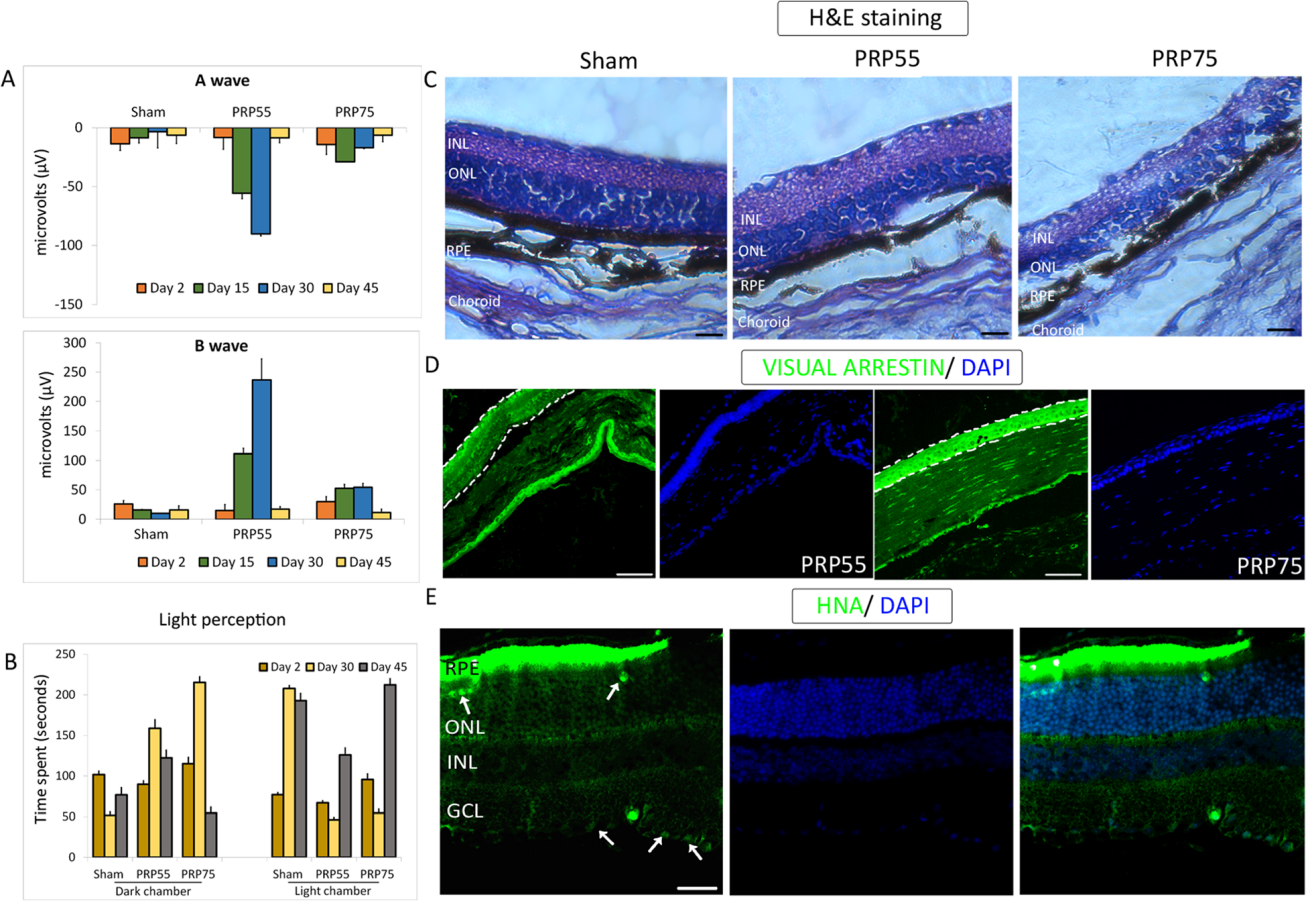
In vivo efficacy studies of PRP transplantation in NOD.SCID-rd1 mice and its readouts. **a** Electroretinography measurement depicted as A wave and B wave for PR55 and PRP75 transplanted in animals. **b** Behavioral study responses with respect to light perception in treated animals. **c** H&E-stained images of Sham, PR55, and PR75 transplanted retina sections at day 45. **d** Immunohistochemical staining of visual arrestin in PR55 and PRP75 transplanted animals. **e** HNA immunostaining shows the presence of human retinal cells in mouse retina after 45 days of transplantation. Scale bars represent 100 μm

In ERG, A wave amplitude indicated that photoreceptor function is a consequence of the electrical response to the light stimulus (Fig. 7a). The B wave amplitude indicated secondary retinal neuronal function (Fig. 7a), which is responsible for the signal transduction via networking neurons such as bipolar and horizontal cells. Both PRP55 and PRP75 groups displayed a transient increase in photoreceptor rescue, thereby indicating a transient enhancement in vision perception, compared with sham controls (Fig. 7b). However, PRP55 exhibited significant functional A wave rescue on days 15 and 30 (Fig. 7a). A wave and B wave displayed similar patterns of improvement in amplitude, indicating functional integration in the neuroretina.

### PRP transplantation furnished transient enhancement in visual perception

The light perception behavioral study measures the visual acuity and perception as mice display photophobia due to their nocturnal nature. Owing to photo-perception, the animal transitions to the dark chamber from the light chamber and displays dark latency due to photo-aversion. The animals transplanted with PRP55 and PRP75 could perceive bright light and, therefore, experience photophobia, spending increasingly more time in the dark chamber from day 2 onwards and most significantly on day 30 until day 45 (Fig. 7b). Post-day 45, light perception was found to deteriorate indicating a decrease in rescue potential. In contrast to the treated animals, sham control animals showed no rescue at any day point and continued to experience loss of perception due to retinal degeneration. In depth perception, animals with visual acuity prefer the shallow side. Both the PRP55 and PRP75 groups showed a preferential transition to the shallow side till day 30, compared with sham controls (Figure S6E). The transition patterns thereby indicate augmentation, albeit short-lived, of visual acuity resulting from functional photoreceptor integration.

### Histology and immunohistochemical staining demonstrated neuroprotection and PRP survival

Hematoxylin and eosin (H&E) staining of paraffin-embedded eye sections was performed to identify morphological changes in mice retina at day 45 post-transplantation (Fig. 7c). The retina of PRP55-transplanted mice showed a small yet noteworthy enhancement in photoreceptor layer thickness compared to sham controls and PRP75-transplanted mice indicating better neuroprotection. Inflammatory cellular infiltration or any other histological changes was not observed. Immunohistochemical staining with visual arrestin also showed increased photoreceptor layer thickness and integrity in the PRP55 group, confirming the H&E results (Fig. 7d). Next, we chose to stain the transplanted sections at day 45 with human-specific nuclear antigen (HNA). At this time point, we found PRP55 human retinal cells integrated between the inner nuclear layer and the ganglion cell layer of the host retina (Fig. 7e). Although the HNA-positive cells were limited, their presence at day 45 post-transplantation is significant. This finding is in line with the transient functional rescue observed with ERG and is indicative of cell survival and integration on day 30 that was not evaluated due to low animal numbers.

## Discussion

After decades of research, pluripotent stem cell-based therapies for retinal disorders have advanced from preclinical studies to phase I/II clinical trials [10, 11, 23, 24]. However, the current gene (Luxturna) or cell therapy products in phase I/II/III trials are projected to cost between USD 150,000 and 450,000, which lays an enormous financial burden on the healthcare system and insurance agencies and is largely unaffordable for a majority of patients. At Eyestem, our long-term vision is to create a scalable and affordable cell therapy platform that can be manufactured with cGMP standards, with a laser focus on producing therapies supported by robust in vivo efficacy and safety.

Here, we present a novel approach that efficiently generated RPE and PRP simultaneously, without employing transwell culture, scaffold, organoids, or mechanical dissection-based enrichment methods. Reportedly, small molecule-mediated retinal induction protocol decreases inconsistency due to variation in lot numbers, manufacturers, and batches of recombinant proteins [25]. Our use of only four additives, three small molecules (IWR1, LDN193189, and SB431542), and one recombinant protein (IGF1), at stage 1 of the differentiation process aligns well with this report. The time course of retinal differentiation with our protocol matched with that in previous reports [26–29]. and iPSCs recapitulated the in vivo milestones of retinogenesis like neuroectoderm, eye field, and optic vesicle/cup stages before giving rise to RPE and PRP. Current cell replacement therapeutic approaches for treating AMD use a dose of 100,000–200,000 RPE cells per eye [23]. The simultaneous yield of RPE (2–3-fold) and PRP (3–4-fold) from a single starting iPSC source using our protocol is fairly high, suggesting that it is both scalable and economically sustainable as an allogeneic cell therapy. cGMP-validated equivalents for the components used in this protocol are commercially available and are currently being tested in our laboratory. Notably, we have also optimized this protocol with human vitronectin (VTN-N), a xeno-free alternative ECM to Matrigel (data not shown).

Whole-genome transcriptomic analysis allowed us to delineate the composition of the cells used for transplantation. Specifically, no endoderm, mesoderm, or central nervous system origin cells were detected post day 70. FOXG1 appears to play an important role in the development of the retina [30], and therefore, the presence of a minor fraction of FOXG1 positive cells in our transplanted RPE cells was not surprising. Likewise, the small percentage of Ki67-positive cells in our RPE cultures is not unusual given the reported proliferative ability of RPE. Such in-depth characterization with respect to identity, purity, potency, and safety of the de novo-generated cells has been lacking to date but is crucial for designing in-process controls and quality checks for manufacturing. Additionally, cryopreservation of cells at intermediate stages permits the pause and restart of differentiation, providing a window for quality assessments. We have identified two potential intermediate stages for cryopreservation—days 20 and 35.

Recently, Lineage Cell Therapeutics Inc. introduced a “thaw and inject” formulation involving hESC-RPE transplantation (OpRegen) for patients with advanced dry AMD as part of their ongoing phase I/II clinical trial [31]. High cell viability and recovery of RPE characteristics in our cells post-freeze-thaw make them suitable for such a “thaw and inject” formulation. However, the efficacy of cell suspension versus RPE sheets has been an ongoing debate [12, 32, 33] and requires further consideration both in terms of therapeutic benefits and feasibility of transport and transplantation/injection of the cellular product.

Survival of the transplanted product in the host tissue is crucial for functional recovery. In RCS rats, the transplanted cells survived well through P90, the latest time point tested, and preserved behavioral measures (OKT) of vision that correlated well with histological indices of photoreceptor protection. A low level of proliferation was observed in the transplanted cells. Although this observation is similar to an earlier report from Astellas [34], we show that these proliferating cells are a subset of the desired RPE population. A small population of proliferative cells may facilitate better survival and engraftment inside the host [35]. Consistent with this, the long-term (day 90) survival of transplanted RPE without any accompanying alarming cell growth and no tumor formations supported the safety profile of the transplanted cells.

A clear inflammatory response was observed in 2 animals that resulted in a compromised cell graft, although this is not uncommon given the xenogeneic nature of the human RPE cells. These human cells when translated to the clinic will be of allogeneic origin. Despite multiple allogeneic cell transplantation studies [9, 36, 37], allogeneic cells still require immunosuppression to survive. Previous papers have documented that human RPE cells may have some inherent immune privileges which minimizes T cell responses, but RPE cells do express other HLA molecules such as HLA-E, which dampens natural killer cells [38, 39].

Compared to other RPE cells generated using other published methodologies that have moved forward to achieve IND approval for early phase clinical trials, our RPE cells appear to have similar or slightly better efficacy [32, 40, 41]. However, more potent comparisons of efficacy are limited by differences in operators of the behavioral parameters among these studies. With respect to photoreceptor preservation and survival, our RPE product appears on par or better than each of the previous products that have achieved IND approval. The efficacy achieved in this study is certainly promising. Studies with a significantly larger “*n*” and multiple longer survival time points are necessary and currently underway.

Neuroprotection and photoreceptor rescue through transplanted photoreceptor progenitors/precursors cells have been shown in the rd1 mouse and Gnat^−/−^ mouse model of night blindness [34, 42]. Very recently, our collaborators demonstrated that peripheral blood-derived monocytes show neuronal properties and integration in immune-deficient rd1 mouse model upon phenotypic differentiation and induction with retinal growth factors [22]. Previously, they reported that while BALB/c displayed an intact retina, CBA/J and NOD.SCID-rd1 lost their outer segment (rod cells) of the photoreceptor layer and ONL in 4 weeks. The thickness of the inner nuclear layer further declined at 8–10 weeks in CBA/J and NOD.SCID-rd1 compared to BALB/c [15]. Significant improvement in ERG responses and light avoidance behavior observed at 30–45 days post-transplantation correlated with the histopathology and visual arrestin staining. Further, demonstration of cell survival using HNA staining is a strong indicator of the integration capacity of hiPSC-derived PRPs. Superior rescue potential with PRP55 over PRP75 could be attributed to variation in the number of surviving cells, difference in characteristics of transplanted cells, or some unknown reasons. Likewise, the decline in vision rescue potential beginning from day 45 post-transplantation could be due to the loss of networking neurons responsible for completing the optical circuit through the formation of ribbon synapses and CNTF secretion.

The interactions between RPE and cells of the neuroretina is crucial for optimal functioning of either cell type. Derivation of pure single cell type populations does not necessarily ensure complete functionality [43]. We propose that a cocktail of cells might better imitate the in vivo interactions. In addition, the hostile environment of the host retina can severely impede graft survival, so a combination of RPE, photoreceptors, and retinal ganglion cells may be more effective for graft survival. We also believe that such an approach will help achieve sustainable therapeutic benefits in case of aggressive, end-stage ONL degeneration [44].

## Conclusions

The success of cell therapy alternatives largely depends on the ease and availability of stem cell-derived cells. Retinal degenerative diseases often present with loss of both RPE and photoreceptors, and the transplantation of both these cells types can address the challenges resulting from disease progression and has a higher chance of vision restoration. The approach used here to generate RPE and PRP cells in parallel is suitable for such combined transplantation strategies. The extensive characterization and the rescue in visual function in rodent models reported in this study confirm the legitimacy of this approach. Based on our proof-of-concept preclinical results, we are currently gearing up to conduct definitive animal efficacy study and GLP safety/toxicology study followed by cGMP manufacturing of hiPSC-RPE cell suspension with the goal of launching a phase 1/2 clinical trial to treat patients with AMD in the near future.

## Supplementary Information

**Additional file 1: **Supplementary methods, tables, and figures. **Table S1.** List of primers. **Table S2.** List of antibodies. **Figure S1:** A) Status of neuro retinal markers in RPE progenitors at day 35- PAX6, Nestin, CRX. B) neural markers GFAPΔ, SOX2. C) stem cell marker OCT4 at RPE day 70, D) proliferation marker Ki67 in day 70 RPE co expressed with MITF and FOXG1. E) Real time PCR data of non-rosette population selected for RPE at days 20, 35, 50, 70 quantified as fold change and expressed as heat map (Left panel).) NGS results provides confirmation on same set of genes in iPSC, retinal progenitors, RPE progenitors, RPE, PRP samples shows clustering and differential expression (Right panel). **Figure S2:** A) Expression of RPE markers in PRP; MITF at day 35, ZO1 at day 35 and day 50. B) Status of neural markers in SOX1, Nestin, FOXG1 and GFAPΔ in day 35 PRP cultures. C) Status of stem cell marker OCT4, proliferation marker Ki67 in day 70 PRP. D) Real time PCR data on rosette selected population destined to form PRP at days 20, 35, 50, 60, 75 quantified as fold change and expressed as heat map. E) KCl (+/-) induced intracellular Calcium imaging of day 75 PRP showed positive response. **Figure S3:** A) Bright field images showing RPE differentiation cultures of an in-house generated iPSC line. B) Expression of RPE markers MITF, PMEL17 and Tyrosinase at day 75. **Figure S4:** A) Primary data score, data quality and read alignment summary. B) Gene Ontology highlighted in retinal progenitors’ stage represented as fold enrichment. C) Differentially up and down regulated genes expressed in different pathways and signaling channels in retinal progenitors, RPE, PRP samples. D) Three sample Venn diagrams showing differentially up and down regulated genes in retinal progenitors, RPE, and PRP. **Figure S5:** OMICS data analysis: Log transformed normalized count from RNAseq data shows clustering and differential expression of iPSC, retinal progenitors, RPE progenitors, RPE, PRP samples represented as heat map. A) Retinal ganglion genes; B) Central Nervous System (CNS) related genes; C) embryonic germ layer- specific genes and D, E) adult RPE and fetal RPE signature genes. F) Significantly dysregulated pathway genes. Heat map color: Red to green through black. **Figure S6:** A) Study design table for RPE animal studies. B) Quantification of histological rescue presented as counted nuclei thick (left) and cones per image (right). C) High dose transplanted animals from P60 and P90 were stained for KI67 and HNA. D) Study design table for PRP animal studies. E) Depth perception behavioral study response in treated animals at different time points.

## Data Availability

mRNA sequencing data has been made available on the Gene Expression Omnibus platform. (https://www.ncbi.nlm.nih.gov/geo/query/acc.cgi?acc=GSE140545).

## Abbreviations

AMD: Age-related macular degeneration
DIM: Differentiation induction media
DPM: Differentiation propagation medium
EBs: Embryoid bodies
ERG: Electroretinography
H&E: Hematoxylin and eosin
HNA: Human-specific nuclear antigen
hESC: Human embryonic stem cell
hiPSC: Human induced pluripotent stem cell
OKT: Optokinetic tracking
ONL: Outer nuclear layer thickness
PRP: Photoreceptor progenitor
PEDF: Pigment epithelium-derived growth factor
RMM: RPE maturation medium
RP: Retinitis pigmentosa
RPE: Retinal pigment epithelium
RCS rats: Royal College of Surgeons rats
VEGF: Vascular endothelial growth factor
