# Supplementary material for: Transplantation of retinal pigment epithelium and photoreceptors generated concomitantly via small molecule-mediated differentiation rescues visual function in rodent models of retinal degeneration
Source: Stem Cell Res Ther. 2021 Jan 19;12:70. doi: 10.1186/s13287-021-02134-x (PMC7814459; doi:10.1186/s13287-021-02134-x)
Supplement: Supplementary file 1 — Additional file 1: Supplementary methods, tables, and figures. Table S1. List of primers. Table S2. List of antibodies. Figure S1: A) Status of neuro retinal markers in RPE progenitors at day 35- PAX6, Nestin, CRX. B) neural markers GFAPΔ, SOX2. C) stem cell marker OCT4 at RPE day 70, D) proliferation marker Ki67 in day 70 RPE co expressed with MITF and FOXG1. E) Real time PCR data of non-rosette population selected for RPE at days 20, 35, 50, 70 quantified as fold change and expressed as heat map (Left panel).) NGS results provides confirmation on same set of genes in iPSC, retinal progenitors, RPE progenitors, RPE, PRP samples shows clustering and differential expression (Right panel). Figure S2: A) Expression of RPE markers in PRP; MITF at day 35, ZO1 at day 35 and day 50. B) Status of neural markers in SOX1, Nestin, FOXG1 and GFAPΔ in day 35 PRP cultures. C) Status of stem cell marker OCT4, proliferation marker Ki67 in day 70 PRP. D) Real time PCR data on rosette selected population destined to form PRP at days 20, 35, 50, 60, 75 quantified as fold change and expressed as heat map. E) KCl (+/-) induced intracellular Calcium imaging of day 75 PRP showed positive response. Figure S3: A) Bright field images showing RPE differentiation cultures of an in-house generated iPSC line. B) Expression of RPE markers MITF, PMEL17 and Tyrosinase at day 75. Figure S4: A) Primary data score, data quality and read alignment summary. B) Gene Ontology highlighted in retinal progenitors’ stage represented as fold enrichment. C) Differentially up and down regulated genes expressed in different pathways and signaling channels in retinal progenitors, RPE, PRP samples. D) Three sample Venn diagrams showing differentially up and down regulated genes in retinal progenitors, RPE, and PRP. Figure S5: OMICS data analysis: Log transformed normalized count from RNAseq data shows clustering and differential expression of iPSC, retinal progenitors, RPE progenitors, RPE, PRP samples represented [file 13287_2021_2134_MOESM1_ESM.docx]

**Supplementary Methods**

**Cryopreservation and revival of RPE and PRP**

RPE and PRP cells were frozen at intermediate (day 20) and late (day 55-75) time points. Cells were washed twice with 1X DPBS and incubated for 3 min at 37^0^C with pre-warmed Accutase. Cells were flushed out from the wells using 1 ml pipette tips and pelleted at 1000 rpm for 2 min at RT. The supernatant was discarded and the cell pellet was resuspended in CryoStor CS10 (Stem Cell technologies) freezing medium at 1 million cells per vial and frozen and stored in liquid nitrogen until further use. When required, the cells were partially thawed in a 37^0^C water bath and immediately transferred to a 15 ml falcon containing sterile media, allowing the cells to completely thaw in the presence of warm media. The cells were pelleted at 1000 rpm for 2 min at RT and the pellet resuspended in appropriate culture media. Cells were seeded onto culture plates at the density of 1 million cells per 10 cm^2^ and media was replenished every other day.

**RNA extraction and RT-PCR**

Total RNA was extracted using Qiagen RNeasy kit. The RNA was converted to cDNA using SuperScript II reverse transcription kit (Thermo Fisher Scientific) per manufacturer’s protocol, and quantitative PCR was performed using Applied Biosystems 7900HT fast Real Time PCR system. Fold change estimations were based on double normalization with β-actin and undifferentiated iPSC. The primers used for PCR are listed in Supplementary Table S1.

**Immunocytochemistry and Flow cytometry**

Cells for immunocytochemistry were seeded in 4-welled chamber dishes and were fixed with 4% paraformaldehyde and stained using a previously described protocol (Surendran et al, Meth Mol Biol 2018). Nuclei were counter stained using DAPI and images were captured using Olympus fluorescent microscope CKX53. Image analysis was performed using ImageJ (NIH) and graphics editing software (Photoshop, Adobe, <https://www.adobe.com>).

Retinal cells were harvested with TrypLE Select to obtain single cell suspension of 1X10^6^ cells that was fixed with 1 ml of 2% paraformaldehyde solution for 15 minutes. Cells were stained with 0.5-1 μg of primary antibody for 20-30 min followed by 0.1 μg of secondary antibody for 15-20 min. The primary and secondary antibodies were diluted in 2% FBS and a 0.1% Triton solution was used for permeabilization when required. Cells were washed to remove any unbound antibody and immediately analyzed on the BD FACS verse machine (BD Biosciences, San Jose, California, www.bdbiosciences.com).For each experiment, a minimum of 10000 events were collected and the study was carried out in biological triplicates. Data analysis was performed using the FlowJo software (FlowJo, Ashland, Oregon, [www.flowjo.com](http://www.flowjo.com)) and quadrant graphs were plotted with respective secondary controls for specific markers. The antibodies used for staining are listed in Supplementary Table S2.

**Quantification of secreted protein by ELISA**

Serum starved culture supernatants of iPSC-derived RPE cells grown in six-welled plates were collected (1.5 mL/well) and aliquots of 100 uL were used to analyze and quantify the secreted vascular endothelial growth factor (VEGF) and pigmented epithelium-derived factor (PEDF) levels using sandwich ELISA method per the manufacturer’s instructions (R&D Systems Inc., Minneapolis, MN, http://www.rndsystems.com). Culture supernatants of ARPE-19 and iPSCs were used as controls.

**Intracellular calcium imaging**

For [Ca2+]i measurements, cells were loaded with 5 uM Fura-2-AM (Molecular Probes, Thermo Fisher Scientific) and 0.002% Pluronic F-127 for 45 min in dark at RT. Culture medium was then changed to recording solution (pH = 7.3) containing 20 mM HEPES, 137 mM NaCl, 5 mM KCl, 2 mM MgCl2, 2 mM CaCl2 and 10 mM D-glucose (Sigma). Cells were incubated for 15 min for complete hydrolyzation of the acetoxymethylester of Fura-2-AM. Ca2+ signals were recorded from single cells using a 60X oil objective (NA = 1.35) after exciting the Fura-2 in the cells at 340 nm and 380 nm (F340 and F380) alternatively, with a time delay of 1 sec in Olympus IX81-ZDC2 Focus Drift Compensating Inverted Microscope. [Ca2+]i was calculated from image data, as described in previous studies. During the experiments, cells were treated with 80 mM KCl. 10 mM Ionomycin (Calbiochem, Merck & co. New Jersey, www.merck.com) was finally added to record the maximum fluorescence values obtained after saturating the dye with Ca2+. Image acquisition was performed using the Andor iXON 897E EMCCD camera and AndoriQ 2.4.2 imaging software (https://andor.oxinst.com).

**RNA sequencing and data interpretation**

Total RNA from RPE and PR cells underwent quality checks and was subjected to directional RNAseq library construction on Illumina HiSeq platform (Illumina, San Diego, California, http://www.illumina.com). For the RNAseq data analysis, the unwanted sequences were removed using Bowtie2 and paired-end reads were aligned using HISAT2 program, which were further used to estimate expression of the transcripts using cufflinks program and reported as FPKM (Fragment per kilo per million) units. Principle component analysis (PCA) and hierarchical clustering was performed with the normalized RNAseq data to find the correlation between the samples. The Differential expression gene (DEG) analysis was performed by calculating the logarithmic fold change in comparison with the iPSC sample. Furthermore, Gene Ontology enrichment analysis was carried out using Amigo2 for DEGs. Pathways classification was performed and overlap was made out of these results using VENNY2.1 to identify a common pool of key genes. Data is represented as heat maps using OMICS data analysis support through XLSTAT add-in feature of Microsoft excel. The RNAseq data has been deposited in Gene Expression Omnibus and is accessible through accession number - GSE140545. (<https://www.ncbi.nlm.nih.gov/geo/query/acc.cgi?acc=GSE140545>)

**Optokinetic Tracking (OKT)**

OKT thresholds were assessed using a virtual optomotor system (VOS; CerebralMechanics) comprising four computer monitors arranged in a square with displays facing inwards. On the monitors, a virtual cylinder was generated that displayed sine-wave gratings that could be rotated either clockwise or counter clockwise permitting evaluation of both the left and right eyes independently; the left eye responds to clockwise and the right eye to counter clockwise movement. On any given trial, the cylinder was centered on the animal’s head effectively clamping the spatial frequency from the animal’s viewing point. Beginning with a low spatial frequency, the cylinder was rotated and if the grating was resolved, the animal then responded with a reflexive head and neck movement tracking the grating. The spatial frequency of the grating was then incrementally increased until the animals no longer tracked the stimulus, resulting in a maximal spatial frequency threshold. Thresholds were evaluated in all live animals on P60 and P90.

**Electroretinography (ERG)**

Mice pupils were dilated with 1% tropicamide and dark adapted for scotopic ERG reading. The animal was then anesthetized using ketamine (80-100 mg/kg) and xylazine (10 mg/kg) intraperitoneally. The gold/active wires, ground electrode, and reference electrode were placed on the cornea, inserted in the tail, and subcutaneously placed between the eyes near the cornea, respectively. The impulse and pulses where standardized by reading the saturation of retinal response on ERG graph. A white light of 10 cds/m^2^ intensity was used to stimulate the whole retina for 25 pulses with 2 msec interval between each pulse. The consequent amplitude of ‘a’ and ‘b’ wave were measured (inbuilt algorithm of LabScribe software). An average of 25 readings was obtained for a single ERG response (MICRON III rodent imaging system using LabScribe software, Phoenix laboratory, USA).

**Behavioral analysis**

**(a) Visual cliff test**

A wooden box of dimensions measuring 62 x 62 x 62 cm (l x b x h) was constructed wherein the four edges emerged at least 19 cm above the top and a wooden platform was placed in the middle dividing the apparatus into equal halves. The platform was 3.75 cm in height, 60 cm in length and 2.5 cm in width. One half of the apparatus was covered with checker paper placed on glass surface giving a perception of shallow side. The other half had the checker board paper on bottom of the apparatus indicated the deep side. The animal was allowed to explore the apparatus for preconditioning prior to starting the experiment. The animal was then placed on the platform and observed for its preference between the sides for 5 min. At half time, the apparatus was turned 180 degree to reduce learning bias. The glass surface was wiped with ethanol to remove familiar cues. The number of transitions to preferred side was counted.

**(b) Light / dark latency test**

A box sized 21 X 42 X 25cm was separated by a partition wall with a 5 cm connecting opening. One half of the chamber was covered with white paper and was brightly lit (approx. 400 lux) while the other half was covered with black paper and was kept dark (below 50 lux). The animal was introduced into light chamber (LC) and observed for 5 min. The total time spent by the animal in each chamber was recorded along with the number of transitions between chambers.

**Histology and immunohistochemistry**

RCS rats were sacrificed using asphyxiation with CO_2_ and by exsanguination, methods approved by the IACUC. At the conclusion of the behavioral testing, rats were euthanized. Both eyes were then removed with the optic nerve, immersion fixed in 4% paraformaldehyde for 24 h, and subsequently infiltrated with 10% sucrose for 1 h, 20% sucrose for 1 h, and 30% sucrose overnight. The following day, both eyes were embedded and frozen in optimum cutting temperature compound (OCT; Tissue Tek #4583, Sakura Finetek, Torrance, https://www.sakuraus.com) adjacent to one another. Sections were collected in a 5-slide series to provide a representative section every 60 µm on each slide throughout the eye-cup. The first slide in each series was stained with cresyl violet or haematoxylin and eosin (H&E). Stained sections were examined for 1) retinal damage/toxicity and 2) evidence of photoreceptor rescue. For each slide, the average outer nuclear layer thickness in temporal and nasal retina was recorded for quantification of photoreceptor rescue. For data presentation purposes, the average of the maximum nuclei thickness is reported. Select remaining slides were then chosen for antibody staining. Light and fluorescent microscopes were used to examine the slides. Antibodies targeted to human RPE cells, including human nuclear antigen and PMEL17 were used to identify the transplanted cells. Cone-arrestin was used to quantify cone photoreceptor rescue. Imaging was done using Olympus IX51 inverted fluorescence microscope under 20X and 40X objectives.

Whole eye was enucleated from the NOD-SCID rd1 mice after euthanasia by cervical dislocation on day 30 and 45 post transplantation. The tissue was washed in PBS once to remove any blood and was immersed in 4% Paraformaldehyde (PFA) for 2 hours at 4^0^C. The fixative was removed by repeated distilled water washes. The tissue was dehydrated with stepwise exposure to ethanol for 1 h each, beginning with 50%, 70%, and then 100% ethanol. Ethanol was then removed by stepwise xylene treatment followed by paraffin replacement. Post treatment, the tissue was embedded in a paraffin block and mounted to cut 4-micron sections of the posterior cup to give retinal sections on poly-L-lysine coated slides. Tissue sections were deparaffinized before staining in coupling jars. Following standard protocol, Haematoxylin stain was added to the sections for 8-10 min to stain the nucleus. Cytoplasm was stained by eosin, and the sections were put in eosin solution (1% solution in distilled water) for 30-60 seconds. Retinal sections were mounted in DPX slide mounting medium (Sigma) and slides were visualized with the help of Olympus CKX53 inverted fluorescence microscope under 20X objective.

**Statistical analysis for animal studies**

All statistical analyses were performed using one way or two way ANOVA post hoc Bonferroni tests. Multi-parametric comparisons between groups were tested with two tailed t-tests. P values less than 0.05 were considered significant.

**Statistical analysis and data availability**

Results are represented as mean+/-standard error (SE) with technical triplicates. Statistical significance was calculated using the Student’s T test - 95% confidence interval and p≤0.05 was considered as statistically significant.

**Supplementary Tables**

**Table S1. List of primers**

| **Gene** | **Primer sequence (5' ---> 3')** |
| --- | --- |
| BESTROPHIN F | GGCAGAACACAAGCAGTTGG |
| BESTROPHIN R | ACGCAAGGTGTTCATCTCGT |
| BLIMP1 F | GTG GTA TTG TCG GGA CTT TG |
| BLIMP1 R | GGT TGC TTT AGA CTG CTC TG |
| BRN3A F | CTCGCTCGAAGCCTACTTTG |
| BRN3A R | GACGCGCACCACGTTTTTC |
| CHX10 F | CGA CAC AGG ACA ATC TTT ACC |
| CHX10 R | CAT AGA CGT CTG GGT AGT GG |
| DCT F | GGTTCCTTTCTTCCCTCCAG |
| DCT R | AACCAAAGCCACCAGTGTTC |
| E-CAD F | GGCACAGATGGTGTGATTACAGTCAAAA |
| E-CAD R | GTCCCAGGCGTAGACCAAGAAA |
| EZRIN F | TCAATGTCCGAGTTACCACCA |
| EZRIN R | AGGCCAAAGTACCACACTTCC |
| LHX2 F | TAC AGG CGC TTC TCT G |
| LHX2 F | GCTCGGGACTTGGTTTATCA |
| LHX2 R | GAT AAA CCA AGT CCC GAG C |
| LHX2 R | GTTGAAGTGTGCGGGGTACT |
| MITF F | CCAGGCATGAACACACATTC |
| MITF R | TCCATCAAGCCCAAGATTTC |
| NESTIN F | CAGCAACTGGCACACCTCAAGAT |
| NESTIN R | AACCAAATGCAGCTTCAGCTTGG |
| NRL F | ATG TGG ATT GGA CGA CTT C |
| NRL R | TTG GCG AGA TTG TCT TGG |
| OCT4 F | GTACTCCTCGGTCCCTTTCC |
| OCT4 R | CAAAAACCCTGGCACAAACT |
| PAX6 F | ATC CGA GAT TTC AGA GCC C |
| PAX6 F | AGTTCTTCGCAACCTGGCTA |
| Recoverin F | CCA GAG CAT GTA CGC CAA CT |
| Recoverin R | CAC GTC GTA GAG GGA GAA GG |
| RPE65 F | GATCTCTGCTGCTGGAAAGG |
| RPE65 R | TGGGGAGCGTGACTAAATTC |
| RX F | GAACAGCCCAAGAAAAAGCA |
| RX R | GCTGTACACGTCCGGGTAGT |
| SOX2 F | CCGCGTCAAGCGGCCCATGAA |
| SOX2 R | GCCGCTTCTCCGTCTCCGACAA |
| SIX3 F | CCGGAAGAGTTGTCCATGTT |
| SIX3 R | CGACTCGTGTTTGTTGATGG |
| THRB F | ACA GGA GAT TTC ATT CGG G |
| THRB R | TTG TAA GAC TAT CAT CTG GGT G |
| TYROSINASE F | ACCCATTGGACATAACCGGG |
| TYROSINASE R | AGAGTCTGGGTCTGAATCTTGT |
| ZO-1 F | TGAGGCAGCTCACATAATGC |
| ZO-1 R | GGGAGTTGGGGTTCATAGGT |
| β-ACTIN F | TCACCCACACTGTGCCCATCTACGA |
| β-ACTIN R | CAGCGGAACCGCTCATTGCCAATGG |

**Table S2. List of antibodies**

| **Primary antibody details** | | | |
| --- | --- | --- | --- |
| **Company** | **Catalogue no.** | **Antigen** | **Specificity** |
| Abcam | ab81213 | BRN3A | Retinal lineage |
| Millipore | MAB5580 | VISUAL ARRESTIN | Retinal lineage |
| GeneTex | GTX124188 | CRX | Retinal lineage |
| Abcam | ab4069 | EZRIN | Early RPE |
| Abcam | ab196868 | FOXG1 | Neural lineage |
| Abcam | ab93251 | GFAPΔ | Neural lineage |
| Abcam | ab15580 | Ki67 | Proliferation |
| Abcam | ab8191 | Ki67 | Proliferation |
| Millipore | ABE1402 | LHX2 | Retinal lineage |
| Abcam | ab59232 | MITF | Early RPE |
| Abcam | ab62734 | NANOG | Pluripotency |
| Abcam | ab221291 | NRL | Rod cell |
| Abcam | ab18976 | OCT4 | Pluripotency |
| Abcam | ab195045 | PAX6 | Neuroectoderm |
| Lifetech | R415 | PHALLOIDIN | F-actin |
| Abcam | ab137078 | PMEL17 | Mature RPE |
| Abcam | ab31928 | RECOVERIN | Cone cell |
| Abcam | ab5417 | RHODOPSIN | Rod cell |
| Abcam | ab13826 | RPE65 | Mature RPE |
| Santacruz | sc271889 | RX | Retinal lineage |
| Abcam | ab109290 | SOX1 | Neuroectoderm |
| Abcam | ab171380 | SOX2 | Neuroectoderm/ Pluripotency |
| Abcam | ab53170 | THRB | Cone cell |
| Abcam | ab16288 | TRA-1-60 | Pluripotency |
| Abcam | ab738 | TYROSINASE | Mature RPE |
| BD biosciences | 610966 | ZO-1 | RPE tight junction |
| BD biosciences | BD610153 | β-CATENIN | Ciliation |
| Millipore | MAB1637 | β-TUBULIN | Neural lineage |
| Millipore | MAB1281 | Anti-Nuclei | Human nuclear antigen |
| **Secondary antibody details** | | | |
| **Company** | **Catalogue no.** | **Antigen** | |
| Abcam | ab150115 | Goat Anti-Mouse IgG H&L (Alexa Fluor® 647) | |
| Abcam | ab150075 | Donkey anti Rabbit IgG H&L [ Alexa Fluor 647] | |
| Abcam | ab150076 | Donkey anti Rabbit IgG H&L [ Alexa Fluor 594] | |
| Abcam | ab150080 | Goat Anti-Rabbit IgG H&L (Alexa Fluor® 594) | |
| Abcam | ab150116 | Goat Anti-Mouse IgG H&L (Alexa Fluor® 594) | |
| Abcam | ab150132 | Donkey Anti-Goat IgG H&L (Alexa Fluor® 594) | |
| Abcam | ab150077 | Goat anti rabbit IgG H&L [Alexa Fluor 488] | |
| Abcam | ab150105 | Donkey Anti-Mouse IgG H&L (Alexa Fluor® 488) | |

**
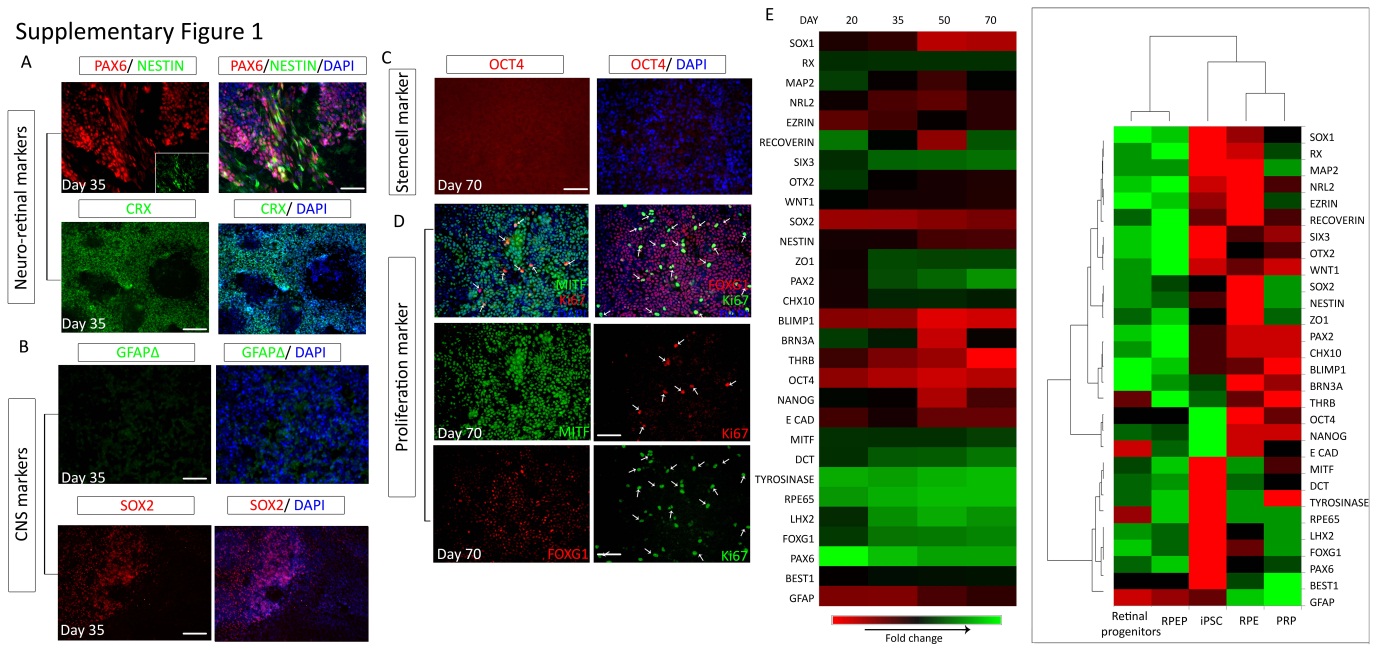
**

**Figure S1:** A) Status of neuro retinal markers in RPE progenitors at day 35- PAX6, Nestin, CRX. B) neural markers GFAPΔ, SOX2. C) stem cell marker OCT4 at RPE day 70, D) proliferation marker Ki67 in day 70 RPE co expressed with MITF and FOXG1. E) Real time PCR data of non-rosette population selected for RPE at days 20, 35, 50, 70 quantified as fold change and expressed as heat map (Left panel).) NGS results provides confirmation on same set of genes in iPSC, retinal progenitors, RPE progenitors, RPE, PRP samples shows clustering and differential expression (Right panel).

**
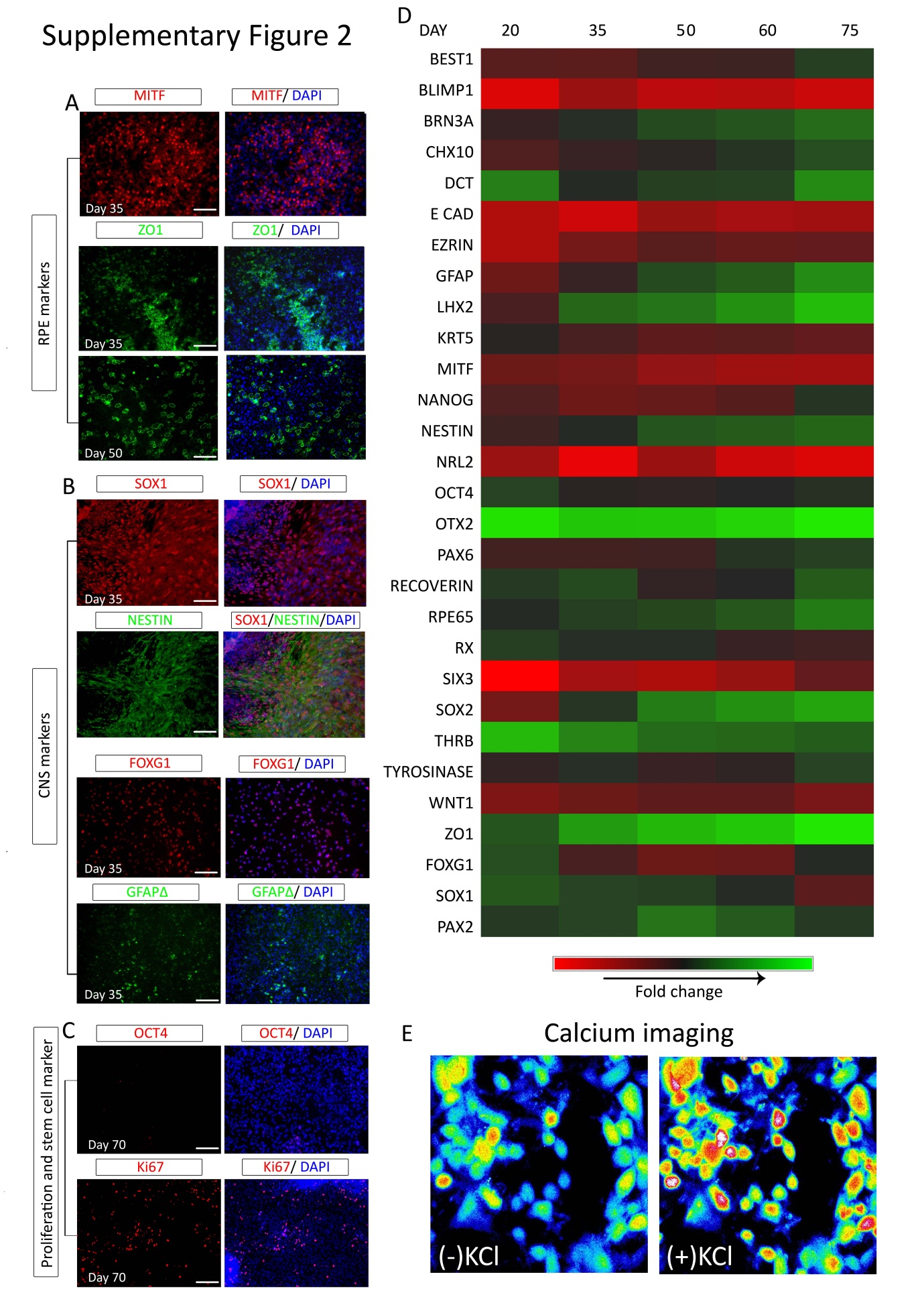
**

**Figure S2:** A) Expression of RPE markers in PRP; MITF at day 35, ZO1 at day 35 and day 50. B) Status of neural markers in SOX1, Nestin, FOXG1 and GFAPΔ in day 35 PRP cultures. C) Status of stem cell marker OCT4, proliferation marker Ki67 in day 70 PRP. D) Real time PCR data on rosette selected population destined to form PRP at days 20, 35, 50, 60, 75 quantified as fold change and expressed as heat map. E) KCl (+/-) induced intracellular Calcium imaging of day 75 PRP showed positive response.


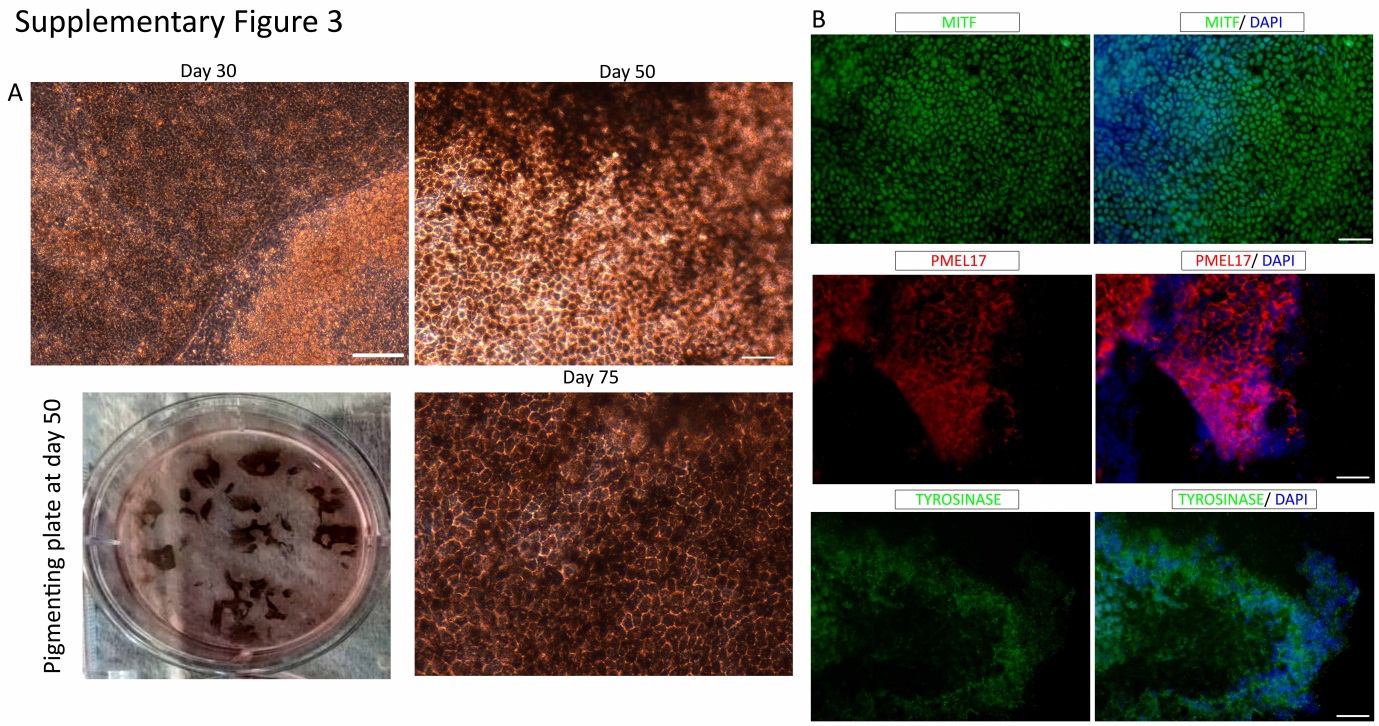


**Figure S3:** A) Bright field images showing RPE differentiation cultures of an in-house generated iPSC line. B) Expression of RPE markers MITF, PMEL17 and Tyrosinase at day 75.

**
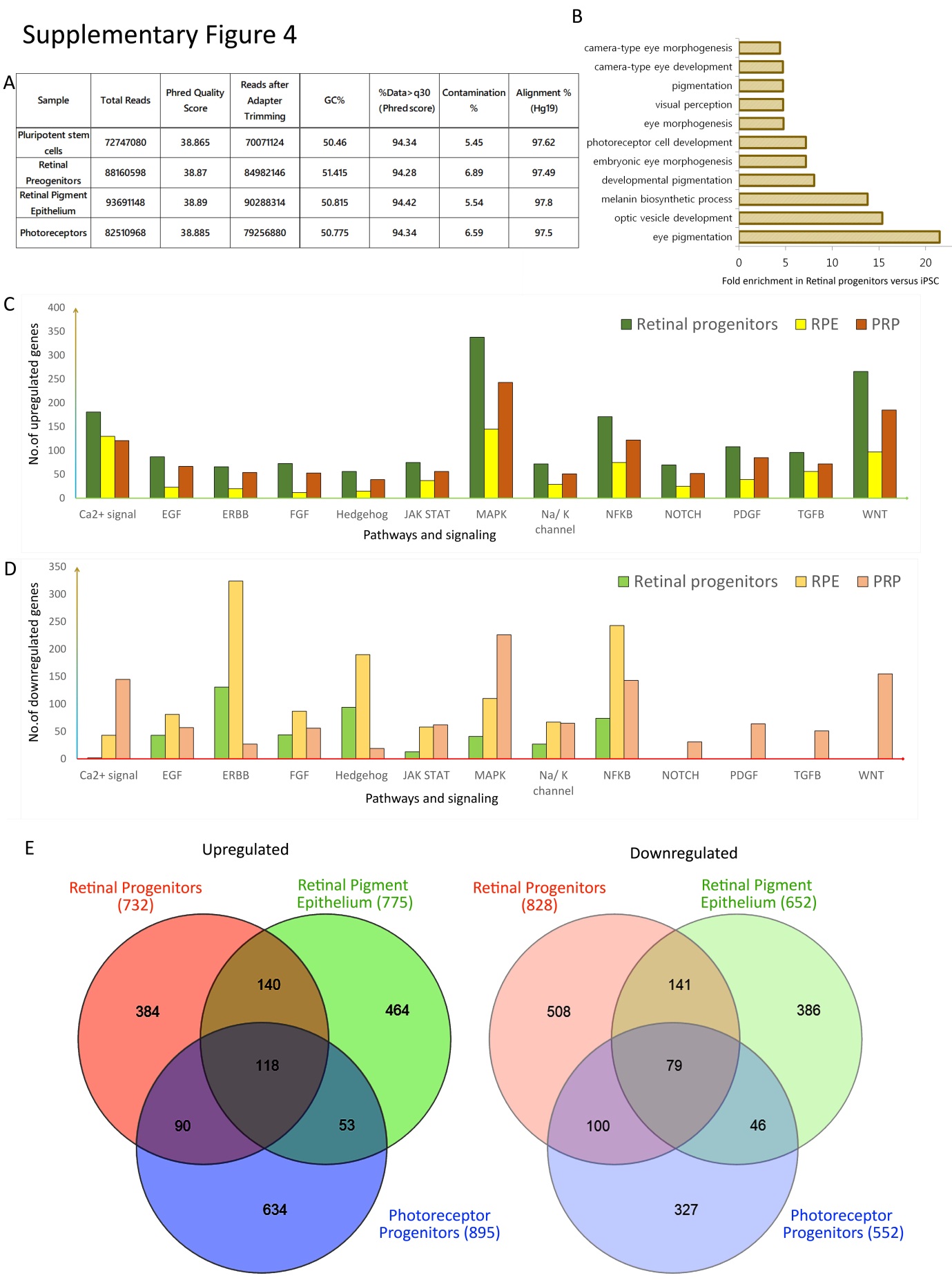
**

**Figure S4:** A) Primary data score, data quality and read alignment summary. B) Gene Ontology highlighted in retinal progenitors’ stage represented as fold enrichment. C) Differentially up and down regulated genes expressed in different pathways and signaling channels in retinal progenitors, RPE, PRP samples. D) Three sample Venn diagrams showing differentially up and down regulated genes in retinal progenitors, RPE, and PRP.

**
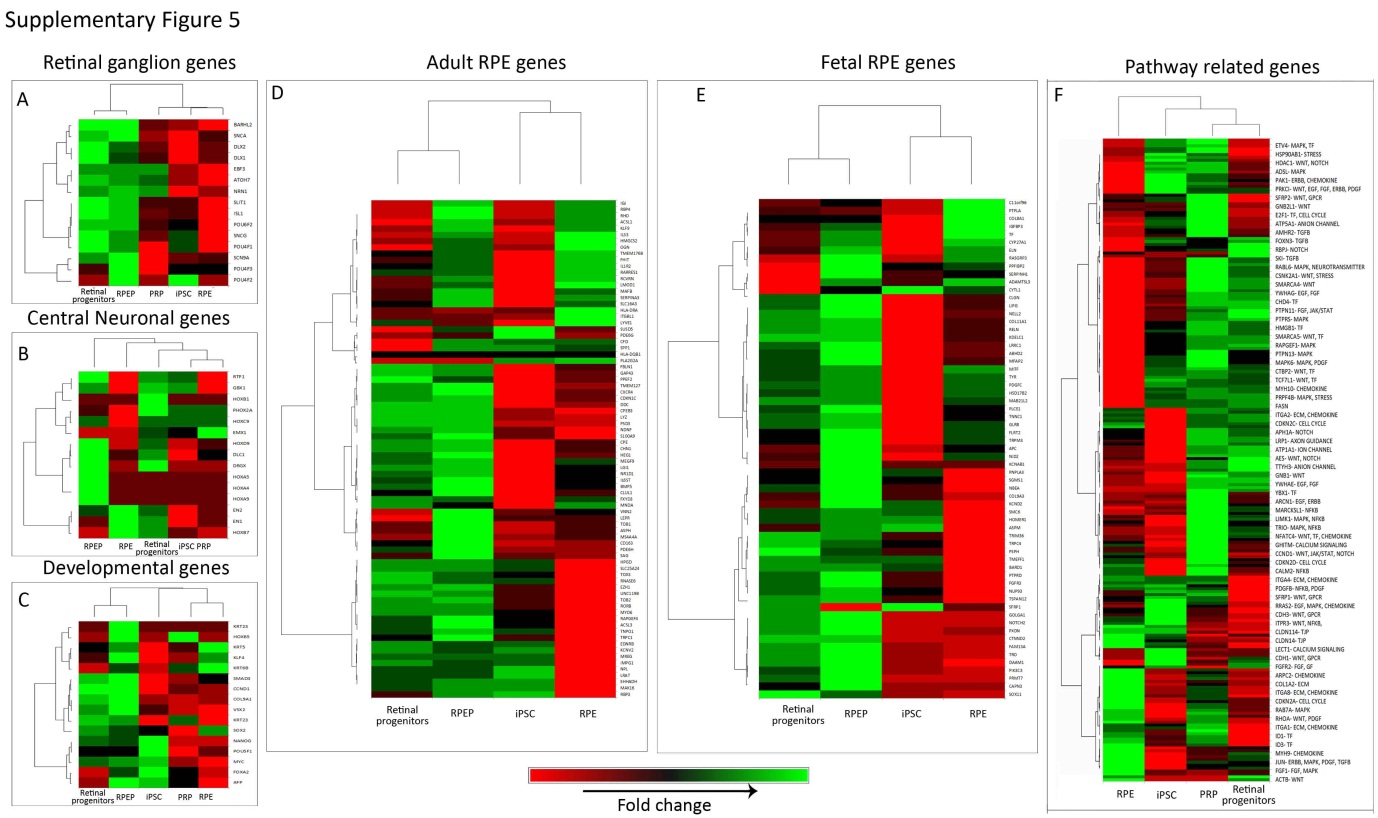
**

**Figure S5:** OMICS data analysis: Log transformed normalized count from RNAseq data shows clustering and differential expression of iPSC, retinal progenitors, RPE progenitors, RPE, PRP samples represented as heat map. A) Retinal ganglion genes; B) Central Nervous System (CNS) related genes; C) embryonic germ layer- specific genes and D, E) adult RPE and fetal RPE signature genes. F) Significantly dysregulated pathway genes. Heat map color: Red to green through black.

**
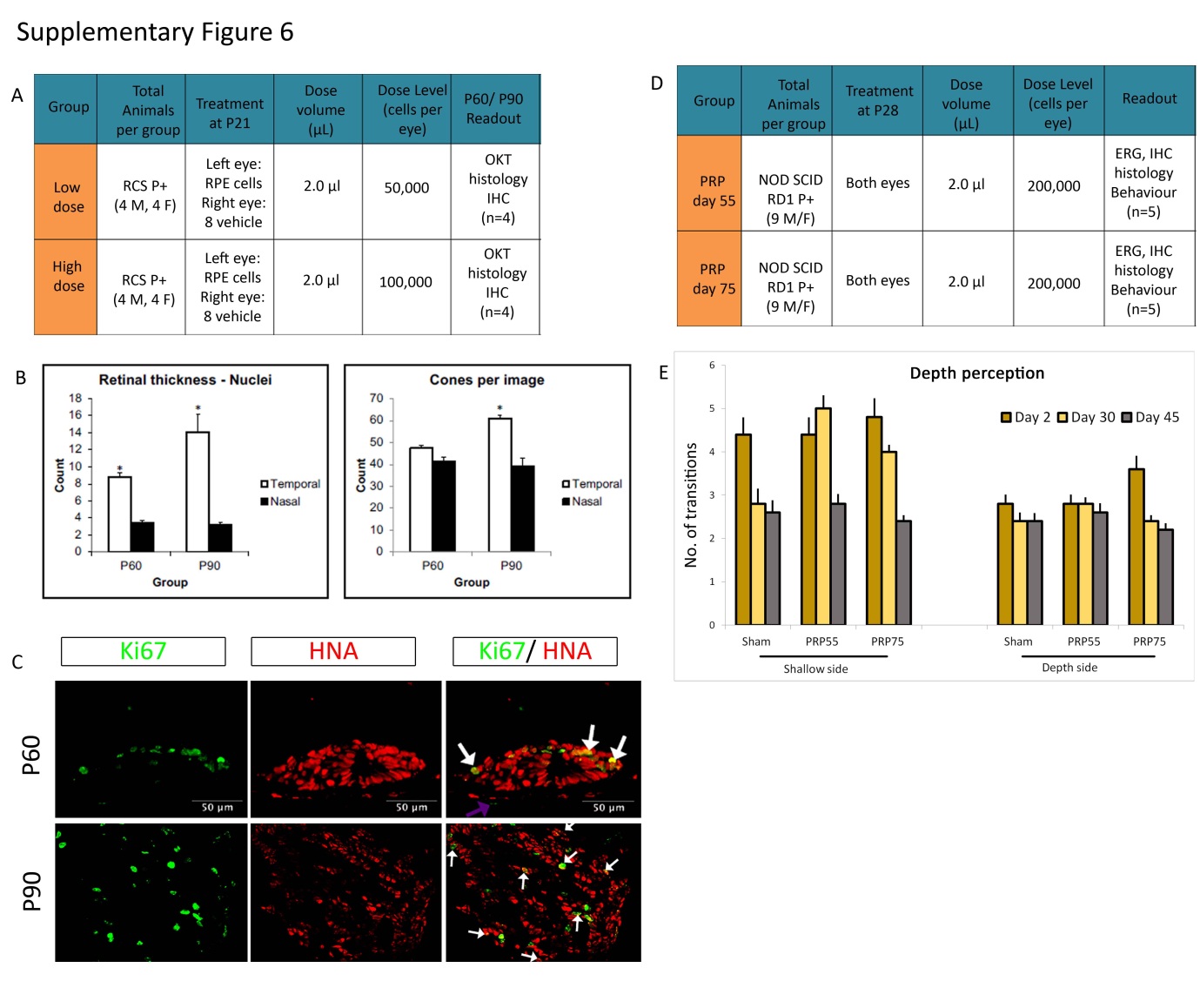
**

**Figure S6:** A) Study design table for RPE animal studies. B) Quantification of histological rescue presented as counted nuclei thick (left) and cones per image (right). C) High dose transplanted animals from P60 and P90 were stained for KI67 and HNA. D) Study design table for PRP animal studies. E) Depth perception behavioral study response in treated animals at different time points.
